# Changes in the gut microbiota of NOD mice in response to an oral *Salmonella*-based vaccine against type 1 diabetes

**DOI:** 10.1371/journal.pone.0285905

**Published:** 2023-05-24

**Authors:** Jacob Cobb, Sameh S. M. Soliman, Mauricio Retuerto, Janine C. Quijano, Chris Orr, Mahmoud Ghannoum, Fouad Kandeel, Mohamed I. Husseiny

**Affiliations:** 1 Department of Translational Research & Cellular Therapeutics, Arthur Riggs Diabetes & Metabolism Research Institute, Beckman Research Institute, City of Hope National Medical Center, Duarte, California, United States of America; 2 Research Institute for Medical and Health Sciences, University of Sharjah, Sharjah, United Arab Emirates; 3 Department of Medicinal Chemistry, College of Pharmacy, University of Sharjah, Sharjah, United Arab Emirates; 4 Center for Medical Mycology, Department of Dermatology, University Hospitals Cleveland Medical Center, Case Western Reserve University, Cleveland, Ohio, United States of America; 5 Faculty of Pharmacy, Zagazig University, Zagazig, Egypt; Mayo Clinic Rochester, UNITED STATES

## Abstract

We developed an oral *Salmonella*-based vaccine that prevents and reverses diabetes in non-obese diabetic (NOD) mice. Related to this, the gastrointestinal tract harbors a complex dynamic population of microorganisms, the gut microbiome, that influences host homeostasis and metabolism. Changes in the gut microbiome are associated with insulin dysfunction and type 1 diabetes (T1D). Oral administration of diabetic autoantigens as a vaccine can restore immune balance. However, it was not known if a *Salmonella*-based vaccine would impact the gut microbiome. We administered a *Salmonella*-based vaccine to prediabetic NOD mice. Changes in the gut microbiota and associated metabolome were assessed using next-generation sequencing and gas chromatography-mass spectrometry (GC-MS). The *Salmonella-*based vaccine did not cause significant changes in the gut microbiota composition immediately after vaccination although at 30 days post-vaccination changes were seen. Additionally, no changes were noted in the fecal mycobiome between vaccine- and control/vehicle-treated mice. Significant changes in metabolic pathways related to inflammation and proliferation were found after vaccine administration. The results from this study suggest that an oral *Salmonella*-based vaccine alters the gut microbiome and metabolome towards a more tolerant composition. These results support the use of orally administered *Salmonella-*based vaccines that induced tolerance after administration.

## Introduction

Type 1 diabetes (T1D) is characterized by autoimmune-mediated destruction of insulin-secreting pancreatic β-cells. Preproinsulin (PPI) is likely one of the diabetes-initiating self-antigens in mice [1] and humans [2–4]. We developed an oral antigen-specific vaccine using live attenuated *Salmonella* [5–7]. The *Salmonella*-based delivery system comprised PPI fused with effector (sseF) and expressed and secreted under control of the SPI2 promoter of the *Salmonella* pathogenicity island 2-encoded type-3 secretion system (SPI2-T3SS) [8–10]. This was combined with the immunomodulators TGFβ and IL10 expressed and secreted under control of a cytomegalovirus (CMV) promoter [5]. The vaccine combined with low dose anti-CD3 mAb prevented and reversed diabetes in non-obese diabetic (NOD) mice [5–7, 11]. *Salmonella* genus belongs to *Enterobacteriaceae* (family), *Enterobacterales* (order), *Gammaproteobacteria* (class), and *Proteobacteria* (phylum) [12] and offers several advantages over the other methods of antigen delivery. *Salmonella* are taken up and internalized by gut antigen presenting cells (APCs) and remain alive in intracellular vacuoles where they pass proteins and genetic information to the host cells [13, 14]. Inside the cells, especially dendritic cells (DCs) and macrophages, the SPI2-T3SS passes proteins directly to the host cytoplasm. Self-antigens fused with SPI2 proteins that are passed directly into the cytosol of APCs can be processed and then presented to immune cells within the gut mucosa [8, 15–18]. Bypassing the expression of antigen in the intestinal lumen has the advantage of avoiding degradation and unwanted immune responses. *Salmonella* can influence host APCs by carrying mammalian expression plasmids and this feature was exploited to develop DNA vaccines [19, 20]. The same feature can be employed to carry tolerogenic cytokines that are directly expressed by the host cell, and hence can create a local immune-privileged microenvironment. Of clinical relevance, a *Salmonella* vaccine for typhoid fever is approved by the FDA.

Since the live *Salmonella* vaccine is orally administered it may alter the microbiota. The gastrointestinal tract microbiota consists of over a 1000 microbial species [21]. The microbiota exists in a state of homeostasis maintained by the action of gut-associated lymphoid tissues (GALTs) that generate regulatory T (Tregs) and effector T cells, B cells, and others. The cross talk between the microbiome and immune cells promotes tolerance. A balanced diverse microbiota was linked to improved digestion, metabolism, and an appropriate immune response to pathogens [22]. Microbiota imbalance, low diversity, and disproportionality are termed dysbiosis. Dysbiosis is associated with various diseases including inflammatory bowel, metabolic, and autoimmune diseases [22, 23].

Imbalance in the gut microbiota was linked to the development of T1D, type 2 diabetes (T2D), and obesity [24]. Moreover, lipopolysaccharide (LPS), as part of the outer membrane of Gram-negative bacteria, possibly derived from gut microbiota, may act as a molecular link between the gut microbiota, inflammation, and T1D. We showed that vaccination using *Salmonella*-based vaccine in combination with sub-therapeutic doses of anti-CD3 antibody reduced the development of diabetes and the severity of insulitis, preserved beta cell mass, and prevented glucose intolerance in mice [5, 6]. Furthermore, combination therapy was capable of reversing ongoing diabetes [7]. In both the prevention and reversal of diabetes, the vaccine significantly increased the number of Tregs in the spleen, mesenteric lymph nodes (MLN), Peyer’s patches (PP) and pancreatic lymph nodes (PLN) [7].

Still, the oral *Salmonella-*based vaccine introduces live bacteria into the gut microbiota. This might have negative consequences on the native intestinal flora. Herein, we explored the effect of the vaccine on the composition and proportions of the gut microbiome and related metabolites in non-diabetic NOD mice.

## Materials and methods

### Salmonella vaccine

The attenuated strain of *Salmonella typhimurium* was employed for oral vaccination as described [5–7, 25]. Bacteria were cultured overnight in liquid growth media containing 50 μg/ml kanamycin or carbenicillin. The cultured bacteria were washed and suspended in 5% sodium bicarbonate for oral administration.

### Animal experiments

Seven-week-old female NOD/ShiLtJ (NOD) mice (Jackson Laboratories, Bar Harbor, ME) were maintained under pathogen-free conditions and housed at the animal care facility at City of Hope National Medical Center. The study was approved by the Institutional Animal Care and Use Committee (IACUC# 18017). Eight-week-old mice were orally vaccinated with *Salmonella* containing plasmid for the expression of IL10 and TFGβ and *Salmonella* expressing autoantigen PPI in 200 ml of 5% sodium bicarbonate on days 0 and 7. Vaccinated animals were also treated with anti-CD3 mAb for five consecutive days (days -1 to 3). The control (vehicle) treatment was 200 ml of 5% sodium bicarbonate given orally. Stools were collected from vaccine-, and control- treated animals at pre, 3-, 7-, 14-, 30-day post-vaccination.

### DNA extraction

DNA was extracted from feces using the QIAamp Fast DNA Stool Mini Kit (Qiagen, Hilden, Germany) [26]. The quality and purity of the isolated genomic DNA were confirmed by gel electrophoresis and quantitated with a Qubit2.0 fluorometer using the Qubit dsDNA HS Assay (Thermo Fisher Scientific, Waltham, MA). DNA samples were stored at -20°C.

### PCR amplification

Amplification of the 16S rRNA and 5.8S rRNA genes were performed using 16S-804 (5′-(TCC TACGGGAGGCAGCAGT-3′), 16S-515 (5′-GGA CTACCA GGG TATCTAATCCTG-3′), ITS1 (5′-TCC GTAGGTGAACCTGCGG-3′), and ITS4 (5′-TCC TCCGCTTATTGATATGC-3′) primers. For identification of fungi, the ITS region was amplified using ITS1 and ITS4 primers and the 16S rRNA gene (V3-V4) was used for bacteria. The PCR mixture was composed of Q5 High-Fidelity Master Mix (New England Biolabs) at a 1x concentration, along with a double volume of molecular grade water and 0.05 μL/mM of each primer. Undiluted DNA (1.5 mL) was added to each 50 mL reaction. Thermocycling conditions included an initial denaturation step (3 minutes at 98°C), followed by 30 cycles of denaturation (10 seconds at 98°C), annealing (10 seconds at 55°C for the16S primers and 20 seconds at 58°C for the ITS primers), extension (10 seconds at 72°C), and a final extension step of 3 minutes at 72°C. PCR products were separated using gel electrophoresis on 1.5% agarose gel.

### Library preparation and sequencing

The amplicon library was cleaned and barcoded, followed by emulsion PCR using the Ion Torrent next-generation sequencing data analysis workflow (Thermo Fisher Scientific). Equal volumes of bacterial 16S rRNA and fungal ITS amplicons were pooled, cleaned with AMPure XP beads (Beckman Coulter, Brea, CA) to remove unused primers, and then exposed to end-repair enzyme for 20 minutes at room temperature. After an additional AMPure cleanup, ligation was performed at 25°C for 30 minutes using Ion Torrent P1 and a unique barcoded “A” adaptor per pooled sample. After AMPure removal of residual adaptors, samples were concentrated to one quarter volume for 1 hour using a vacuum (Labconco, Kansas City, MO) under heat. All separate barcoded samples were then pooled in equal amounts (10 μL) and size-selected for the anticipated 16S and ITS range (200 to 800 bp) using Pippin Prep (Sage Bioscience, Beverly, MA). The library was amplified for 7 cycles and quantitated on a StepOne qPCR instrument (Thermo Fisher Scientific) ahead of proper dilution to 300 pM going into the IonSphere templating reaction on the Ion Chef (Thermo Fisher Scientific). Library sequencing was completed on an Ion Torrent S5 sequencer (Thermo Fisher Scientific), and barcode-sorted samples were analyzed in our custom pipeline based on GreengenesV13_8 and Unite database V7.2, designed for the taxonomic classification of 16S rRNA and ITS sequences, respectively. Sequencing reads were clustered into operational taxonomic units (3% distance), described by community metrics and taxonomically classified within the Qiime bioinformatics pipeline (ver. 1.9)

### Metabolomics profiling

Metabolomic profiling was performed as published [27]. Feces (20 mg) was mixed with methanol: chloroform (1:1) and vortexed for 15 minutes. Samples were centrifuged at 10,000 RPM for 10 minutes and the filtrates were collected and dried using a rotatory evaporator (Buchi, Germany). The metabolite extracts were derivatized by adding 50 μL of *N*-trimethylsilyl-*N*-methyl trifluoroacetamide and trimethylchlorosilane (MSTFA + 1% TMS) mixture followed by incubation at 50˚C for 30 minutes prior to gas chromatography-mass spectrometry (GC-MS) analysis as described [28]. GC-MS measurements were carried out using an Agilent Model 7683 Autosampler, 6890 Gas Chromatograph and 5975 Inert Mass Selective Detector in the Electron Impact (EI) mode. EI energy was set to 70 eV. Separation was carried out on an Agilent HP5-MS column with dimensions 30 m x 250 μm x 0.25 μm. Ultra-High Purity Grade He (Airgas) was used as the carrier gas with the flow set to 0.8 mL/minute in constant flow mode. The initial oven temperature was set to 45°C for 1 minute followed by a 30°C/minute ramp to a final temperature of 300°C, which was maintained for 3 minutes. A 3.2-minute solvent delay was used. The injector temperature was set at 220°C. The MSD was set to scan the 40–1050 m/z range. Data collection and analysis were performed using MSD Enhanced Chemstation software (Agilent). Product spectra were identified by comparison of the measured fragmentation patterns to those found in the NIST 08 Mass Spectral Library.

Differential metabolite expression was calculated as the fold-change from day 0 (before vaccination) by employing Ingenuity Pathway Analysis (IPA, Qiagen, version 84978992) core analysis with metabolomics analysis by expression Log Ratio using default settings and no cutoffs. Assessment between timepoints was created using the comparison analysis tool in IPA. Analyzed pathway data was exported into CSV (comma-separated values) files and graphed in Prism.

### Statistical analysis

A custom pipeline based on the Greengenes V13_8 database designed for the taxonomic classification of 16S rRNA sequences was employed. Downstream data analysis was performed using Qiime Platform (ver. 1.9) [29]. The Dunnett’s multiple comparisons test and Dunn’s multiple comparisons test (non-parametric) were used to analyze the differences in the percent abundance of gut microbiome within the same cohorts before and after vaccination (paired for longitudinal analysis). Bonferroni’s and Mann-Whitney multiple comparisons tests were used to compare differences between vaccinated and vehicle-treated groups at different time points. Multiple paired t tests with adjusted p-values using the two-step Benjamini, Kreiger, and Yekutiele procedure were used to compare differences in post-vaccinated metabolite concentrations in animals on day 3, 7, 14, and 30 with pre-vaccinated levels. For all tests, a p < 0.05 was considered significant. Statistics were performed using Graphpad Prism software.

## Results

### Microbiota analysis

Fifty fecal samples were collected from vaccine- (n = 25) and control (vehicle)-treated NOD mice. The total number of bacteriome and mycobiome reads was 3.7 million. Annotated sequences were used to study the microbial community using the phylotype approach. Sequences were assembled in operational taxonomic units (OTUs) at the lowest annotation level (genus). The relative abundance for each OTU was calculated. The resulting abundance matrix was used to calculate the community alfa diversity indexes. Relative abundances of bacterial and fungal phyla, genera, and species are represented in **S1** and **S2 Figs**.

### Effect of oral *Salmonella*-based combination therapy on gut bacteriome

To answer the question if the oral bacterial vaccine alters the gut bacterial community composition, the difference between vaccine- and vehicle-treated groups was assessed before and after vaccination on days 3, 7, 14, and 30 post-vaccination. The dynamics of the murine gut microbiota was studied at the levels of phylum and genus.

**Fig 1:**
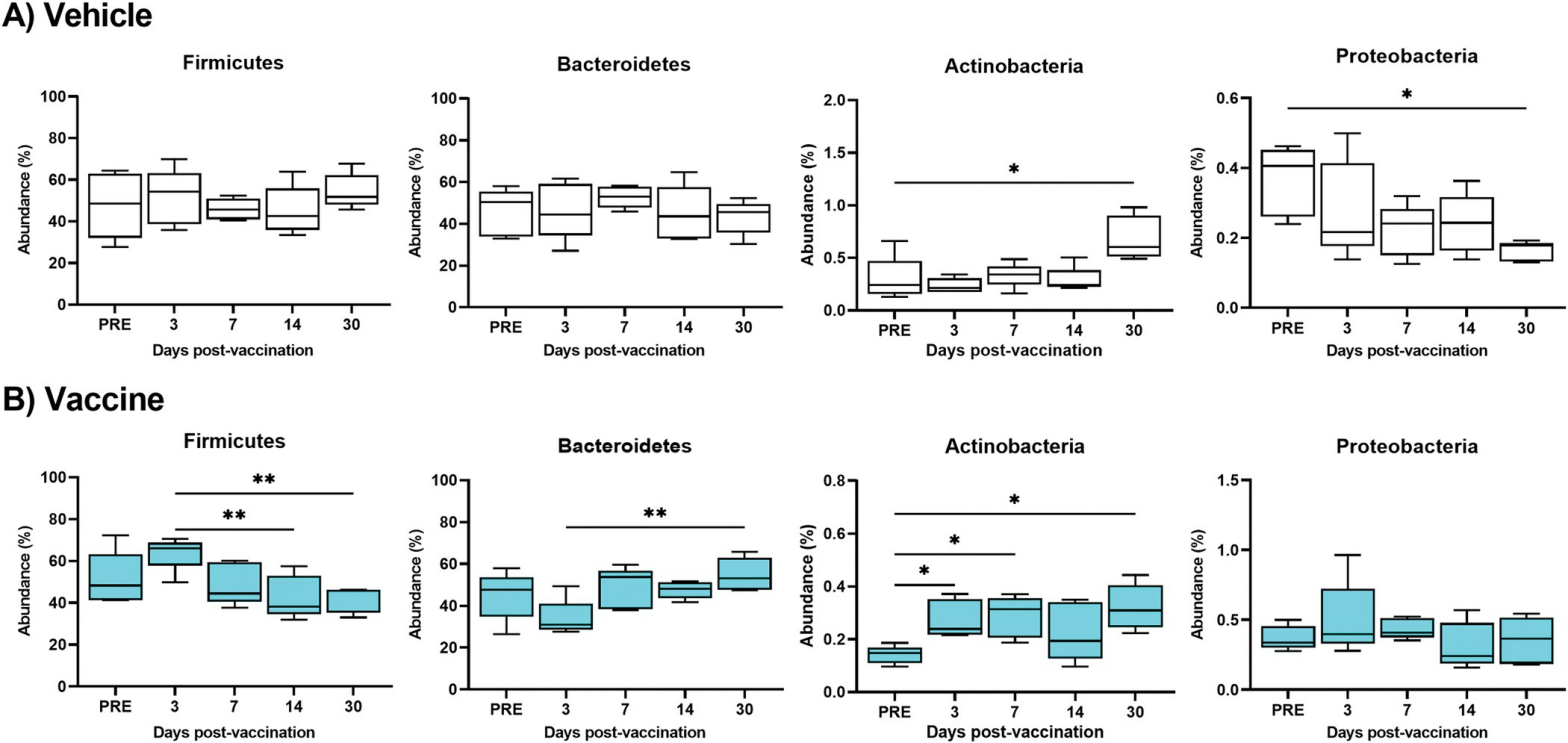
Boxplots of changes in abundance percentage of the gut bacteria at the phylum level within the same treated cohorts at different time points. The longitudinal analysis of 4 most abundant phyla including *Firmicutes*, *Bacteroidetes*, *Actinobacteria*, and *Proteobacteria* are shown within vehicle cohorts (A), and vaccine cohorts (B). Each time point represents the mean ± SD of 5 samples. The Dunnett’s multiple comparisons test was used to analyze the significance between different time points using asterisks, *p < 0.05 and **p < 0.01.

The murine gut microbiota was dominated by two bacterial phyla *Firmicutes* and *Bacteroidetes* that accounted for over ~ 95% of sequence reads at all time points (**S3A Fig**). The remaining diversity was attributed to *Verrucomicrobia*, *Tenericutes*, *Actinobacteria*, and *Proteobacteria* that accounted for ~ 4% of the remaining microbiota abundance. The phyla with abundance less than 1% were combined and designated “other” (**S3A Fig**). Longitudinal analysis of the fecal bacterial composition was conducted. At the phylum level, a significant decrease in *Firmicutes* on days 14 and 30 (p = 0.005) (**Fig 1B**) was observed for vaccine-treated mice, while no change in the *Firmicutes* was found in vehicle-treated mice (**Fig 1A**). *Bacteroidetes* was decreased on day 3 and significantly increased on day 30 (p = 0.009) (**Fig 1B**) in vaccine-treated mice, but not in vehicle-treated mice (**Fig 1A**). *Actinobacteria* was significantly increased in vaccine-treated mice on days 3, 7 and 30 (p = 0.01, p = 0.02, and p = 0.02, respectively) (**Fig 1B**), while a significant increase in *Actinobacteria* on day 30 (p = 0.028) was observed in vehicle-treated mice (**Fig 1A**). No change was detected in fecal *Protobacteria* in vaccine-treated mice (**Fig 1B**), while in vehicle-treated mice a significant decrease in *Protobacteria* (p = 0.011) was noted (**Fig 1A**).

**Fig 2:**
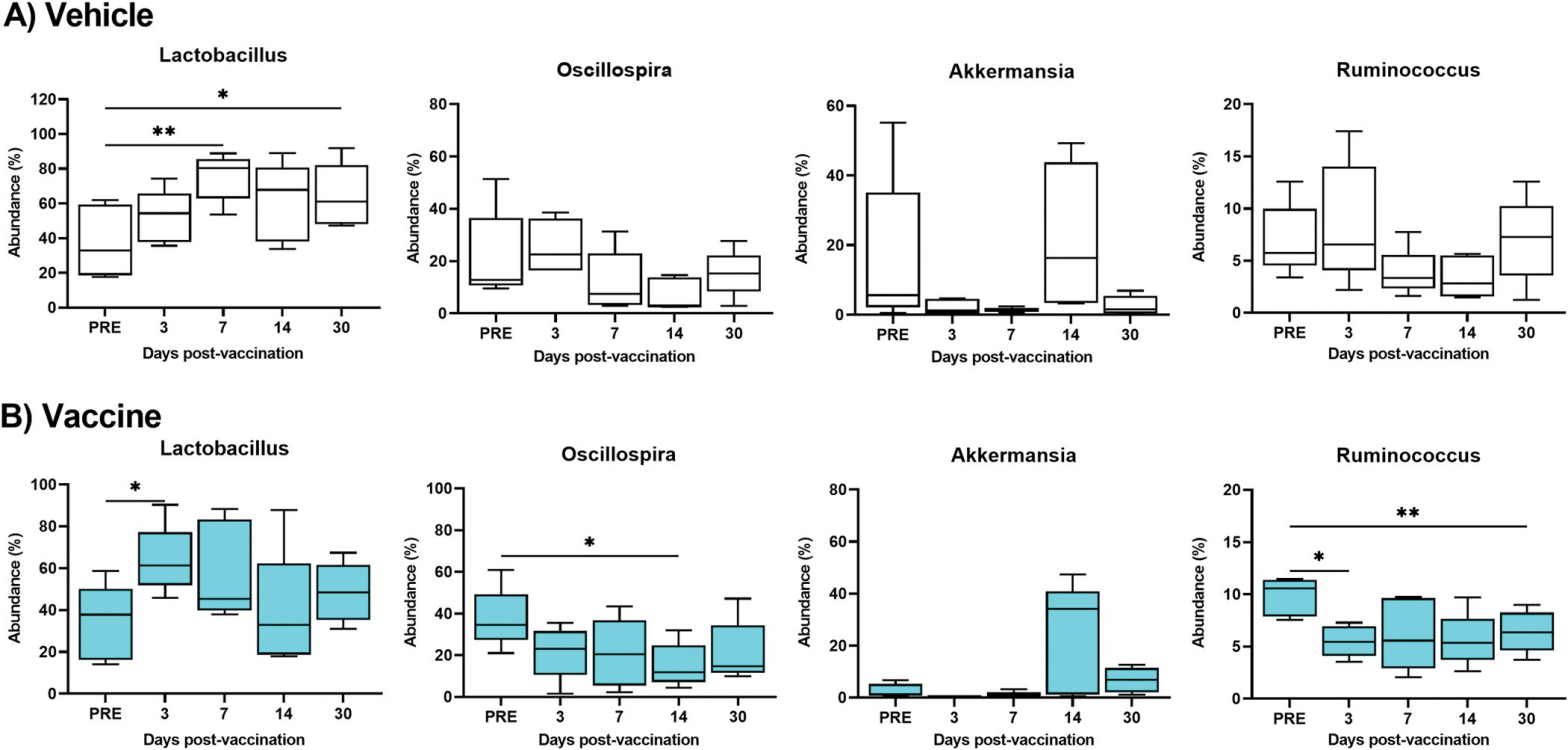
Boxplots of changes in abundance percentage of the gut bacteria at the genus level within the same treated cohorts at different time points. The longitudinal analysis of the 4 most abundant genera including *Lactobacillus*, *Oscillospira*, *Akkermansia*, and *Ruminococcus* are shown within vehicle cohorts (A), and vaccine cohorts (B). Each time point represents the mean ± SD of 5 samples. The Dunnett’s multiple comparisons test was used to analyze the significance between different time points using asterisks, *p < 0.05, and **p<0.01.

The genera levels of microbial dynamics are displayed in **S3B Fig**. Ten genera with abundance >1% are presented. The genera with abundance <1% were combined and designated “other” (**S3A Fig**). There genera dominated, *Lactobacillus*, *Oscillospira*, and *Akkermansia* accounting for ~ 75%. In longitudinal analysis, *Lactobacillus* was significantly increased on day 3 (p = 0.043) in vaccine-treated mice (**Fig 2B**) and significantly increased on days 7 (p = 0.007) and 30 (p = 0.034) in vehicle-treated mice (**Fig 2A**). The genus *Oscillospir*a was significantly decreased on day 14 (p = 0.012) (**Fig 2B**) in vaccine-treated animals and little changed in vehicle-treated mice (**Fig 2A**). The genus *Ruminococcus* was significantly decreased on days 3 (p = 0.04) and 30 (p = 0.007) (**Fig 2B**) but decreased without significance in vehicle-treated mice (**Fig 2A**). No change in the abundance of *Akkermansia* in any of the mice was observed (**Fig 2**).

**Fig 3:**
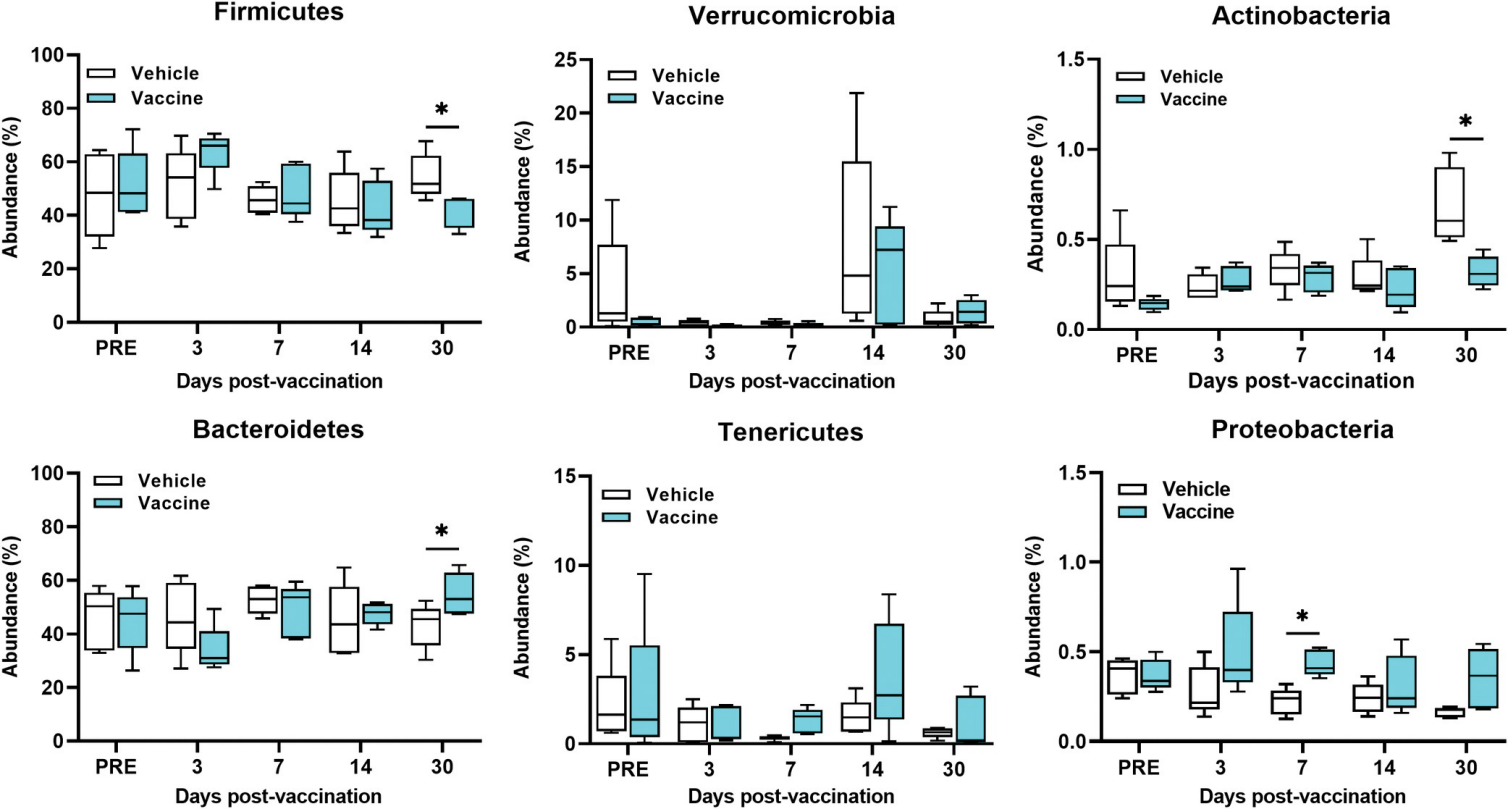
Boxplots of changes in abundance percentage of the gut bacteria at the phylum level. The 6 most abundant phylum including *Firmicutes*, *Bacteroidetes*, *Verrucomicrobia*, *Tenericutes*, *Actinobacteria*, and *Proteobacteria* are shown. Each time point represents the mean ± SD of 5 samples. Mann-Whitney with Holm-Sidak correction for multiple comparisons test was used to report significance between groups at each time point using asterisks, *p < 0.05.

At the phylum level, changes over the 5 time points for the 6 most abundant fecal bacteria in vaccine-treated mice compared with the vehicle-treated mice are illustrated in **Fig 3.** The relative abundance of the phylum *Bacteroidetes* was significantly increased in the gut of vaccine-treated mice on day 30 (p = 0.024) and on day 7 (p = 0.03) for *Proteobacteria* compared with vehicle-treated mice (**Fig 3**). In contrast, the abundances in the bacterial phyla *Firmicutes* and *Actinobacteria* in vaccine-treated mice were significantly decreased on day 30 (p = 0.023, and p = 0.008) compared with vehicle-treated mice (**Fig 3**). No changes were observed in the relative abundance of the bacterial phyla *Verrucomicrobi*a and *Tenericutes* between vaccine- and vehicle-treated groups at any time points (**Fig 3**).

**Fig 4:**
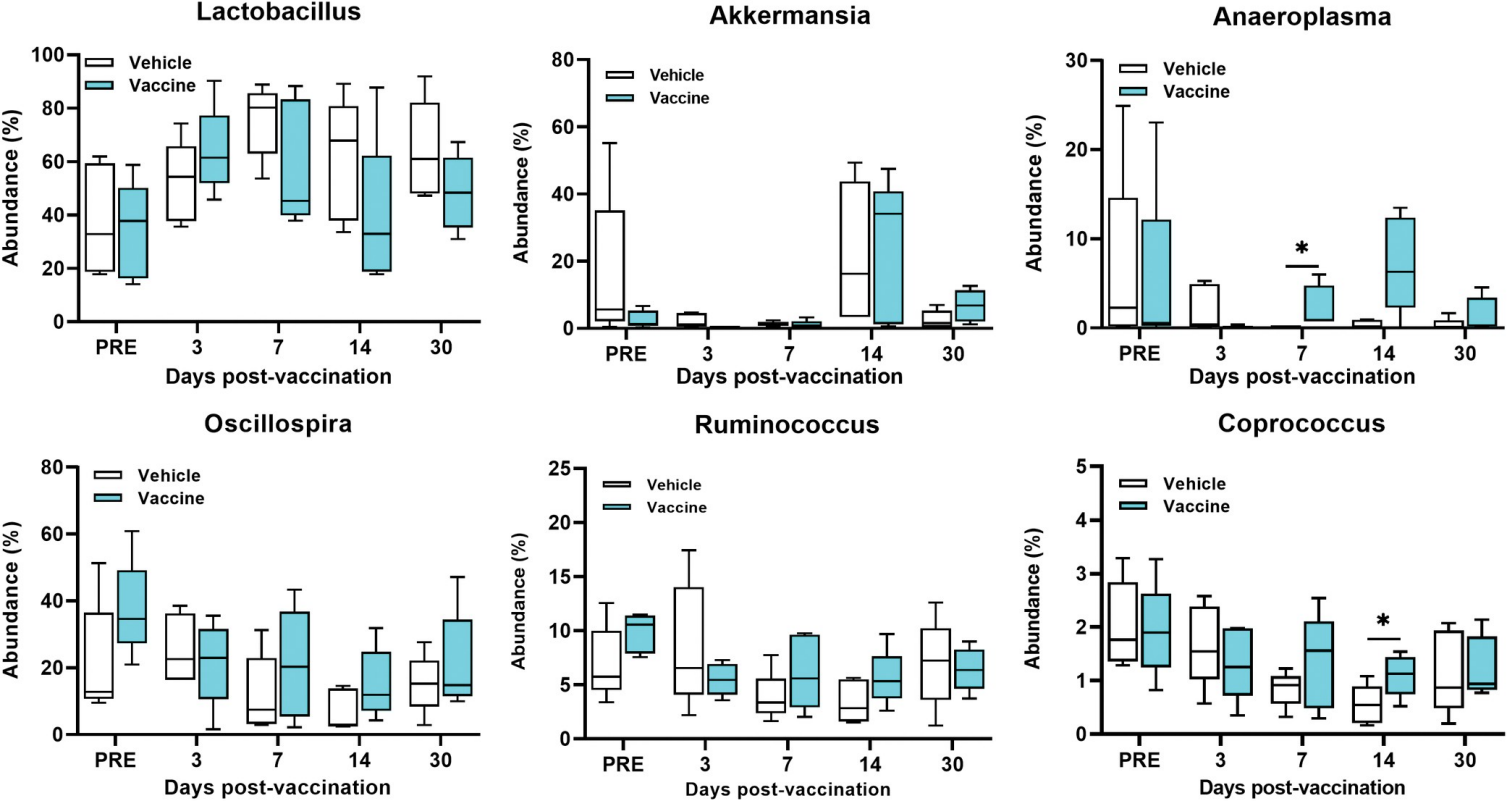
Boxplots of changes in the abundance percentage of the fecal bacteria at the genus level. The 6 most abundant genera including *Lactobacillus*, *Oscillospira*, *Akkermansia*, *Ruminococcus*, *Anaeriplasma*, and *Coprococcus* are shown. Each time point represents the mean ± SD of 5 samples. Mann-Whitney with Holm-Sidak correction for multiple comparisons test was used to report significance between groups at each time point using asterisks, *p < 0.05.

No significance changes occurred in the 6 most abundant fecal bacterial compositions at the genus level over any time points except a significant increase in the genus *Anaeroplasma* on day 7 (p = 0.008) and *Coprococcus* on day 14 (p = 0.03) in vaccine- compared with vehicle-treated mice (**Fig 4**).

**Fig 5:**
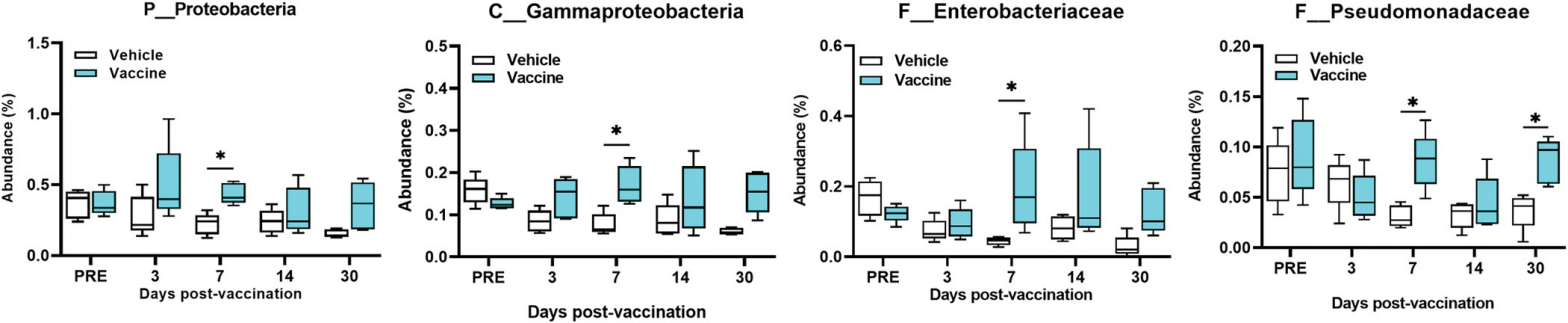
Boxplots of the changes in abundance percentage of the fecal selected phylum *Proteobacteria*. The percentage of class *Gammaproteobacteria*, families *Enterobacteriaceae* and *Pseudomonadaceae*, and genus *Pseudomonas*. Each time point represents the mean ±SD of 5 samples. Group of vaccinated mice compared with vehicle-treated at different time points. Mann-Whitney with Holm-Sidak correction for multiple comparisons test was used to report significance between groups at each time point using asterisks, *p < 0.05.

There is a relation between *Salmonella* and phylum *Protobacteria*. Thus, a closer look into this was done. A significant increase in the phylum *Protobacteria* (relative abundance 0.5%) on day 7 (p = 0.029) occurred in vaccine-treated mice compared to vehicle-treated mice (**Fig 5**). The class *Gammaproteobacteria* (~0.3%) significantly increased on day 7 in vaccine- compared to vehicle-treated mice (p = 0.019) (**Fig 5**). Analyzing the class *Gammaproteobacteria* including families *Enterobacteriaceae* (0.3%) and *Pseudomonadaceae* (0.1%) revealed a relative abundance of fecal bacteria from *Enterobacteriaceae* with a significant increase noted in vaccine- compared to vehicle-treated on day 7 (p = 0.022) (**Fig 5**). Also, a significant increase in the relative abundance of the bacterial family *Pseudomonadaceae* was noted in vaccine- compared to vehicle-treated mice on days 7 (p = 0.008) and 30 (p = 0.008) (**Fig 5**).

### Effect of oral *Salmonella*-based combination therapy on the gut mycobiome

The mouse gut mycobiome is dominated by the fungal phylum *Ascomycota* that accounted for over 99% of sequence reads (**S4A Fig**). The remaining detected phyla included *Basidiomycota*, *Zygomycota*, and *Glomeromycota*. No significant difference in the fecal fungal phyla between vehicle- and vaccine-treated animals at any time points (**S4A Fig**). No statistically significant differences in the 4 most abundant fecal fungi at the phylum level were noted across any time points between vaccine- and vehicle-treated mice (**S5A Fig**).

The composition of gut fungal genera of mouse feces was dominated by 4 genera. *Galactomyce*s, *Eurotium*, *Candida*, and *Geotrichum* accounted for ~90% (**S4B Fig**). No statistically significant differences in the 4 most abundant fecal fungal genera were noted across all time points between vaccine- and vehicle-treated mice (**S5B Fig**).

In summary, vaccination had no significant effect on gut microbiota composition immediately after initiation of treatment. The effects were noted at later times and were characterized by the increased abundance of the phylum *Bacteroidetes* and decreased abundance in *Firmicutes* and *Actinobacteria*.

### Effects of an oral *Salmonella*-based combination therapy on the gut metabolome

Gut metabolic profiling was conducted on feces collected from NOD mice at pre- and post-vaccination days 3, 7, 14, and 30. Metabolomic profiling showed variation in the metabolites between samples (**Table 1**). The major metabolites identified were fatty acids such as palmitic acid, myristic acid, oleic acid, stearic acid, 6-octadecenoic acid (petroselaidic acid), α-linolenic acid, 1,4-butanediol, 1-pentadecanol, butanoic acid, pentadecanoic acid, and heptadecanoic acid; amino acids such as L-leucine, L-isoleucine, L-valine, L-tyrosine, L-alanine, and phenylalanine; and sugars such as β-D-(+)-xylopyranose, glycerol, L-rhamnose, D-ribose, α-d-glucopyranoside, in addition to urea, and 3,5-dihydroxybenzoic acid (**Table 1**).

**Table 1:** Metabolites detected in the faces of NOD mice before and after vaccination using GC-MS.

Metabolite	Before vaccination		After vaccination											
	PRE		Day 3			Day 7			Day 14			Day 30		
	Average	±SD	Average	±SD	P #	Average	±SD	P #	Average	±SD	P #	Average	±SD	P #
Urea	ND	-	0.06	0.02	**-**	13.99	0.83	**-**	0.62	0.22	**-**	12.32	0.41	**-**
Uracil	0.87	0.03	0.34	0.08	**0.011**	0.24	0.06	**<0.001**	1.38	2.25	**0.019**	0.42	0.06	**0.004**
L-Leucine	0.37	0.12	0.26	0.11	**0.454**	0.56	0.07	**0.225**	0.59	0.16	**0.553**	0.44	0.10	**0.062**
L-Isoleucine	0.47	0.19	0.29	0.05	**0.274**	0.52	0.08	**0.944**	0.52	0.09	**0.807**	0.39	0.08	**0.358**
L-Tyrosine	0.43	0.07	0.37	0.01	**0.238**	0.52	0.11	**0.325**	0.66	0.13	**0.110**	0.45	0.05	**0.748**
L-Valine	0.38	0.05	0.19	0.07	**0.101**	0.43	0.09	**0.309**	0.55	0.09	**0.052**	0.42	0.04	**0.165**
Glycine	0.05	0.02	0.05	0.06	**0.942**	0.03	0.01	**0.626**	0.03	0.02	**>0.999**	0.02	0.01	**0.374**
DL-Alanine	1.415	0.22	0.10	0.08	**0.117**	0.83	0.24	**0.245**	1.24	0.17	**0.449**	1.07	0.18	**0.509**
L-Phenylalanine	0.36	0.07	ND	-	**-**	0.47	0.08	**0.321**	0.52	0.08	**0.075**	0.48	0.07	**0.152**
5-Oxoproline (Pyroglutamic acid)	0.99	0.15	0.46	0.11	**0.078**	0.51	0.07	**0.266**	0.79	0.13	**0.218**	0.43	0.06	**0.013**
Butanedioic acid (Succinic acid)	0.50	0.10	0.41	0.07	**0.093**	0.39	0.07	**0.363**	0.51	0.11	**0.371**	0.37	0.06	**0.190**
Oxalic acid	0.10	0.09	0.14	0.03	**0.775**	0.07	0.01	**0.517**	0.31	0.40	**0.960**	0.08	0.01	**0.658**
Glycolic acid (hydroacetic acid)	0.58	0.09	0.59	0.26	**0.762**	0.55	0.05	**0.500**	0.81	0.12	**0.123**	0.59	0.15	**0.461**
Butanoic acid (Ethyl acetic acid)	0.43	0.20	0.83	0.25	**0.232**	ND	-	**-**	0.82	0.04	**0.088**	0.14	0.04	**0.350**
5-Aminovaleric (Propylacetic) acid	0.32	0.24	0.52	0.11	**0.106**	1.20	0.27	**0.034**	0.88	0.14	**0.046**	0.69	0.17	**0.087**
3-Hydroxybutyric acid (BHBA)	0.52	0.01	0.25	0.15	**0.439**	0.06	0.01	**0.175**	0.09	0.01	**0.475**	ND	-	**-**
Lactic (2-Hydroxypropanoic) acid	2.72	0.58	7.18	0.89	**0.004**	2.80	0.55	**0.299**	4.68	0.71	**0.060**	2.54	0.59	**0.735**
3-Hydroxypropanoic (Ethylene lactic) acid	0.57	0.24	0.41	0.12	**0.246**	0.42	0.10	**0.361**	0.77	0.13	**0.200**	0.45	0.07	**0.365**
Hexadecanoic acid, ethyl ester (Palmitic) acid	0.66	0.04	0.79	0.16	**0.656**	0.51	0.11	**0.395**	0.96	0.12	**0.076**	0.94	0.18	**0.334**
Palmitic Acid	8.95	1.92	9.75	1.22	**0.325**	5.49	1.65	**0.035**	9.27	1.07	**0.368**	12.58	1.22	**0.161**
Myristic acid	1.30	0.33	1.00	0.25	**0.602**	ND	-	**-**	0.71	0.12	**0.043**	0.69	0.19	**0.024**
Octanoic acid (Caprylic acid)	0.24	0.06	0.15	0.04	**0.075**	ND	-	**-**	0.30	0.14	**0.462**	0.22	0.06	**0.960**
Pentadecanoic acid	0.58	0.13	0.81	0.19	**0.238**	0.72	0.04	**0.097**	0.67	0.16	**0.449**	0.78	0.16	**0.085**
Stearic acid	1.66	0.49	2.12	0.46	**0.086**	1.24	0.33	**0.299**	1.81	0.43	**0.993**	2.37	0.44	**0.025**
Heptadecanoic (Margaric) acid	0.51	0.31	0.280	0.06	**0.206**	0.20	0.04	**0.166**	0.24	0.08	**0.256**	0.29	0.06	**0.258**
1-Tetradecanol	0.45	0.27	0.683	0.09	**0.162**	0.30	0.12	**0.889**	0.50	0.06	**0.747**	0.50	0.06	**0.693**
10-Undecynoic acid	0.88	0.11	0.663	0.15	**0.342**	0.58	0.06	**0.038**	0.10	0.01	**0.042**	0.66	0.09	**0.190**
Oleic Acid	1.10	0.07	0.170	0.06	**0.007**	1.20	0.56	**0.214**	1.11	0.13	**0.959**	0.54	0.04	**0.005**
α -Linolenic acid	ND	-	1.070	0.06		ND	-	**-**	ND	-	**-**	0.92	0.42	**-**
9,12-Octadecadienoic (Linoleic) acid	4.82	0.68	4.880	0.46	**0.931**	3.14	0.24	**0.268**	2.24	0.66	**0.079**	5.12	0.75	**0.620**
Azelaic acid	0.49	0.01	0.342	0.11	**0.006**	0.35	0.09	**0.135**	0.74	0.12	**0.105**	0.44	0.08	**0.605**
Ethanolamine	2.00	0.34	1.125	0.21	**0.112**	0.65	0.09	**0.007**	ND	-	**-**	0.81	0.11	**0.011**
4-Hydroxybenzeneacetic acid	0.64	0.17	0.533	0.06	**0.498**	0.547	0.09	**0.336**	0.74	0.26	**0.823**	0.56	0.07	**0.497**
3-(3-Hydroxyphenyl) propanoic acid	1.57	0.46	3.203	0.13	**0.025**	1.51	0.31	**0.934**	2.39	0.97	**0.244**	2.33	0.80	**0.163**
Phloretic (3-(4-Hydroxyphenyl) propanoic) acid	0.34	0.02	0.587	0.15	**0.330**	0.22	0.05	**0.170**	0.55	0.10	**0.085**	0.37	0.09	**0.747**
Eicosane	0.43	0.11	0.964	0.14	**<0.001**	0.84	0.06	**<0.001**	1.98	0.63	**0.005**	0.59	0.07	**0.001**
Heptadecane	0.43	0.09	0.246	0.04	**0.028**	0.22	0.06	**0.068**	0.62	0.11	**0.078**	0.16	0.03	**0.011**
Cholesterol	0.36	0.04	0.630	0.08	**0.061**	0.50	0.06	**0.088**	0.56	0.11	**0.154**	0.58	0.07	**0.022**
Cholestan-3-ol (Coprostanol)	0.13	0.02	0.158	0.04	**0.020**	0.13	0.01	**0.580**	0.10	0.06	**0.625**	0.15	0.03	**0.011**
24-Ethyl-.delta.(22)-Coprostenol	0.04	0.01	0.080	0.01	**0.060**	0.05	0.01	**0.423**	0.24	0.21	**0.235**	0.08	0.02	**0.082**
Stigmastanol	0.24	0.06	0.166	0.04	**0.067**	0.15	0.01	**0.019**	0.25	0.02	**0.547**	0.23	0.03	**0.724**
Campesterol	0.24	0.05	0.182	0.05	**0.123**	0.19	0.02	**0.242**	0.30	0.05	**0.218**	0.26	0.03	**0.949**
Stigmasterol	0.12	0.04	0.095	0.01	**0.369**	0.07	0.02	**0.344**	0.15	0.03	**0.286**	0.08	0.01	**0.306**
β.-Sitosterol	0.10	0.02	0.120	0.01	**0.074**	0.09	0.02	**0.500**	0.12	0.01	**0.321**	0.09	0.01	**0.184**
D-Ribose	0.65	0.20	0.540	0.21	**0.568**	ND	-	**-**	ND	-	**-**	0.76	0.04	**0.933**
L-Rhamnose	0.49	0.42	1.020	0.06	**0.012**	ND	-	**-**	0.23	0.01	**0.431**	0.80	0.34	**0.298**
Xylose	0.21	0.03	0.243	0.05	**0.529**	0.18	0.09	**0.795**	0.25	0.04	**0.156**	0.23	0.05	**0.423**
β- D-(+)-Xylopyranose	0.94	0.57	0.958	0.08	**0.951**	ND	-	**-**	0.65	0.08	**0.503**	0.90	0.10	**0.920**
D-(+)-Talofuranose	0.29	0.06	0.275	0.12	**0.971**	0.27	0.05	**0.950**	0.26	0.06	**0.701**	0.29	0.05	**0.669**
Levoglucosan	1.68	0.65	0.267	0.04	**0.184**	0.88	0.04	**0.315**	0.32	0.08	**0.216**	0.16	0.04	**0.170**
Glucopyranose	1.00	0.09	0.724	0.17	**0.082**	0.86	0.12	**0.012**	0.44	0.07	**0.008**	0.76	0.18	**0.056**
Methyl α.-D-glucofuranoside	2.14	0.55	2.475	0.47	**0.122**	1.16	0.26	**0.007**	1.29	0.30	**0.023**	1.11	0.41	**0.010**
N-Acetyl-D-galactosamine	0.30	0.09	0.437	0.05	**0.267**	0.31	0.05	**0.858**	0.60	0.10	**0.050**	0.26	0.03	**0.203**
Glyceric acid	0.81	0.10	1.026	0.28	**0.454**	0.76	0.34	**0.907**	0.97	0.50	**0.482**	1.18	0.44	**0.041**
Glycerol	0.85	0.22	1.047	0.33	**0.590**	4.84	1.86	**0.110**	**ND**	-	**-**	2.29	0.67	**0.022**
1-Monooleoylglycerol	0.26	0.09	ND	-	**-**	0.16	0.05	**0.336**	0.15	0.07	**0.089**	ND	-	**-**
Glycerol monostearate	1.78	0.28	1.880	1.16	**0.928**	1.24	0.10	**0.006**	1.59	0.47	**0.829**	1.61	0.36	**0.351**
1-Monopalmitin (Glyceryl palmitate)	0.40	0.06	0.610	0.06	**0.088**	0.21	0.05	**0.004**	0.35	0.05	**0.396**	0.38	0.07	**0.714**
1,3-Propanediol	0.15	0.05	0.150	0.03	**0.500**	0.09	0.02	**0.053**	0.02	0.01	**0.025**	0.10	0.03	**0.597**
2,6-Bis (tert-butyl) phenol	2.59	0.40	1.616	0.27	**0.017**	ND	-	**-**	1.63	0.59	**0.132**	1.72	0.30	**0.028**

The amount represents the relative percentage of a metabolite in relation to total metabolites detected in the fecal pellet for 5 mice before and on days 3, 7, 14, and 30 post-vaccination ± SD. The statistical significance was calculated using multiple paired *t* test with Benjamini, Kreiger, and Yekutiele corrected significance level indicated (p value < 0.05). Red p value means significant increase and blue p value means significant decrease in comparison to the pre-treated group.

**Fig 6:**
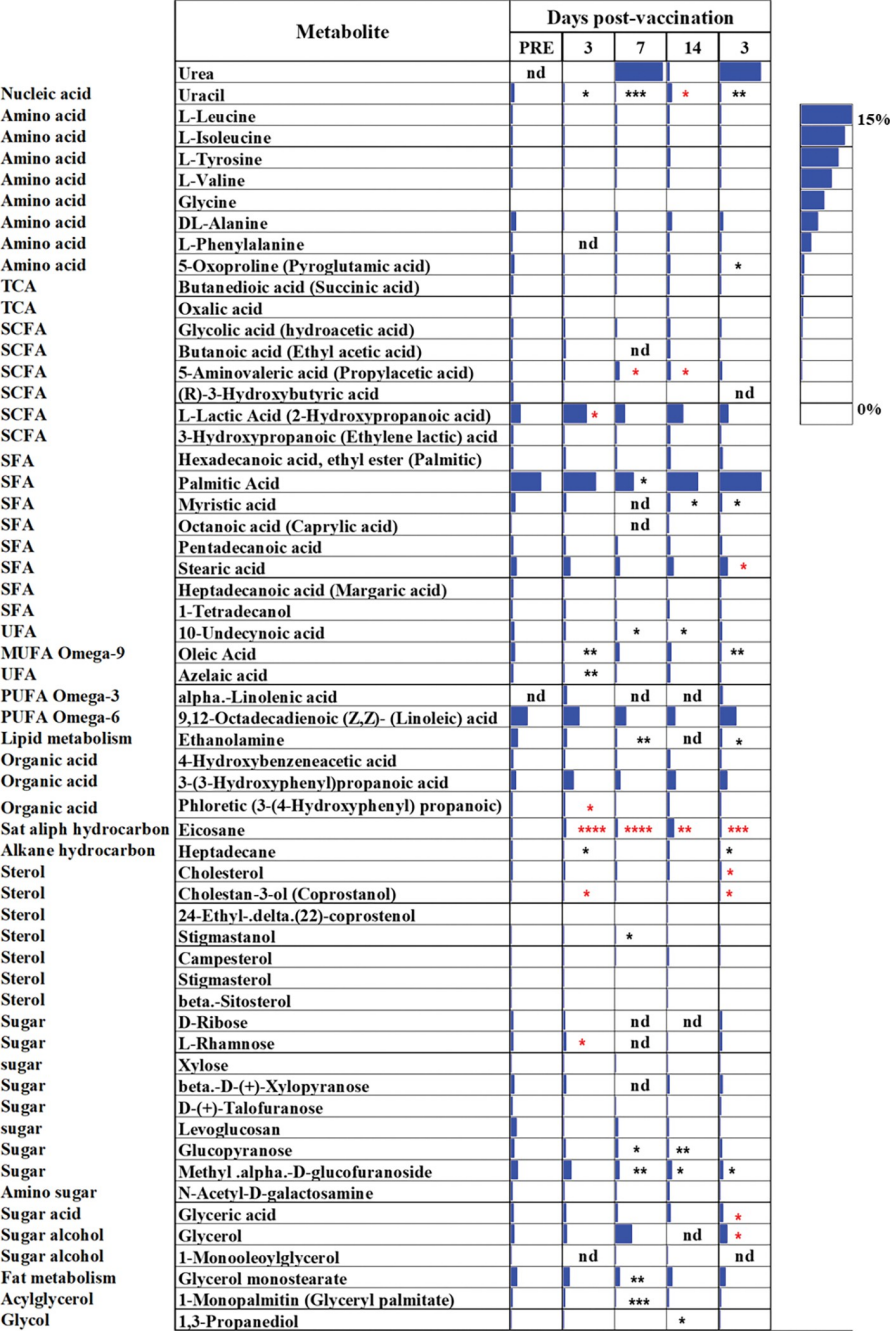
Heatmap comparing the metabolites extracted from feces of NOD mice at different times point pre- and post-vaccination using GC-MS. Feces collected from mice before and after vaccination at days 3, 7, 14, and 30. The amount represents the relative percentage of a metabolite in relation to total metabolites detected in a fecal pellet. Metabolite average relative percentages of 5 mice at each time point are displayed as blue bars. The statistical significance was calculated using multiple paired *t* test with Benjamini, Kreiger, and Yekutiele corrected significance level indicated asterisks (*p < 0.05, **p<0.01, ***p<0.005, ****p<0.001). Red asterisks mean a significant increase and black asterisks mean a significant decrease in comparison to the pre-treated group. SCFA (short-chine fatty acid); SFA (saturated fatty acid); UFA (unsaturated fatty acid); MUFA (monounsaturated fatty acid); PUFA (polyunsaturated fatty acid); TCA (tricarboxylic acid cycle); nd (not detected).

Some of these metabolites showed specific patterns following vaccination. L-leucine was significantly decreased in the feces following vaccination on days 3 and 7 and then significantly increased on day 14 and dropped again on day 30 while L-5-oxoproline (pyroglutamic acid) was decreased after vaccination and more so on day 30. Other amino acids including L-isoleucinem, L-valine, L-alanine, L-tyrosine, glycine, and phenylalanine were varied (**Fig 6**).

The short-chain fatty acids (SCFA) derivative 5-aminovaleric acid (propylacetic acid) was significantly increased on days 7 and 14 while L-lactic acid (2-hydroxypropanoic acid) was significantly increased (day 3) after vaccination then returned to normal. No effect was noted on glycolic acid (hydroacetic acid) and 3-hydroxypropanoic (ethylene lactic) acid while 3-hydroxybutyric acid was decreased after vaccination (**Fig 6**).

Saturated fatty acids (SFA) including myristic acid on days 14 and 30 and palmitic acid at day 7 were significantly decreased in feces following vaccination. On the other hand, stearic acid was significantly increased on day 30 in feces following vaccination while other SFA such as hexadecanoic acid ethyl ester (palmitic acid), pentadecanoic acid, and heptadecanoic acid (margaric acid) varied but without significant changes (**Fig 6**).

Unsaturated fatty acids (UFA) such as 10-undecynoic acid (at days 7 and 14) and oleic acid (on days 3 and 30) were significantly decreased following vaccination while α-linolenic acid was detected only on days 3 and 30 following vaccination (**Fig 6**). Interestingly, azelaic acid as a product of oleic acid was significantly decreased in the feces on day 3 following vaccinations while no changes were noted in the 9,12-octadecadienoic acid (linoleic acid) (**Fig 6**).

Other acids such as 3-(3-hydroxyphenyl) propanoic acid (3-hydroxycinnamic acid) was significantly increased in the feces at day 3 following vaccination and then returned to normal levels on day 30. Other metabolites such as phloretic acid (3-(4-hydroxyphenyl) propanoic acid) and 4-hydroxybenzeneacetic acid were not changed in the feces after vaccination while ethanolamine was significantly decreased on days 7 and 30 after vaccination. Eicosane, a saturated hydrocarbon, was significantly increased after vaccination while heptadecane was significantly decreased (**Fig 6**).

Sugar metabolites including levoglucosan (on day 30), and glucopyranose, and methyl alpha-D-glucofuranoside were significantly decreased in the feces following the vaccination, while D-ribose, D- (+)-talofuranose, xylose, beta-D-(+)-xylopyranose and N-acetyl-D-galactosamine did not change in the feces after vaccination. L-rhamnose was significantly increased on day 7 in the feces after vaccination (**Fig 6**).

Glycerol monostearate (2-monostearin) and glyceryl palmitate (1-monopalmitin) were significantly decreased on day 7 following vaccination and returned to normal on day 14, and 1,3-propanediol was significantly decreased on day 14. Glycerol and glyceric acid were increased after vaccination and become significantly increased on day 30 (**Fig 6**).

Steroid metabolites detected in feces such as cholesterol (on day 30) and cholestane-3-ol (coprostanol) were significantly increased on days 3 and 30 after vaccination while stigmastanol was significantly decreased on day 14. Others such as stigmasterol, β-sitosterol, and campesterol 24-ethyl-delta (22)-coprostenol were not changed after vaccination (**Fig 6**).

Fecal metabolites such as succinic acid (butanedioic acid) and oxalic acid were not changed following vaccination (**Fig 6**). Urea was detected in the feces of the animal on day 3 then sharply increased on day 7 followed by a decrease on day 14 and then a final increase on day 30 post-vaccination while uracil (a nucleic acid) was significantly decreased following vaccination (**Fig 6**).

**Fig 7:**
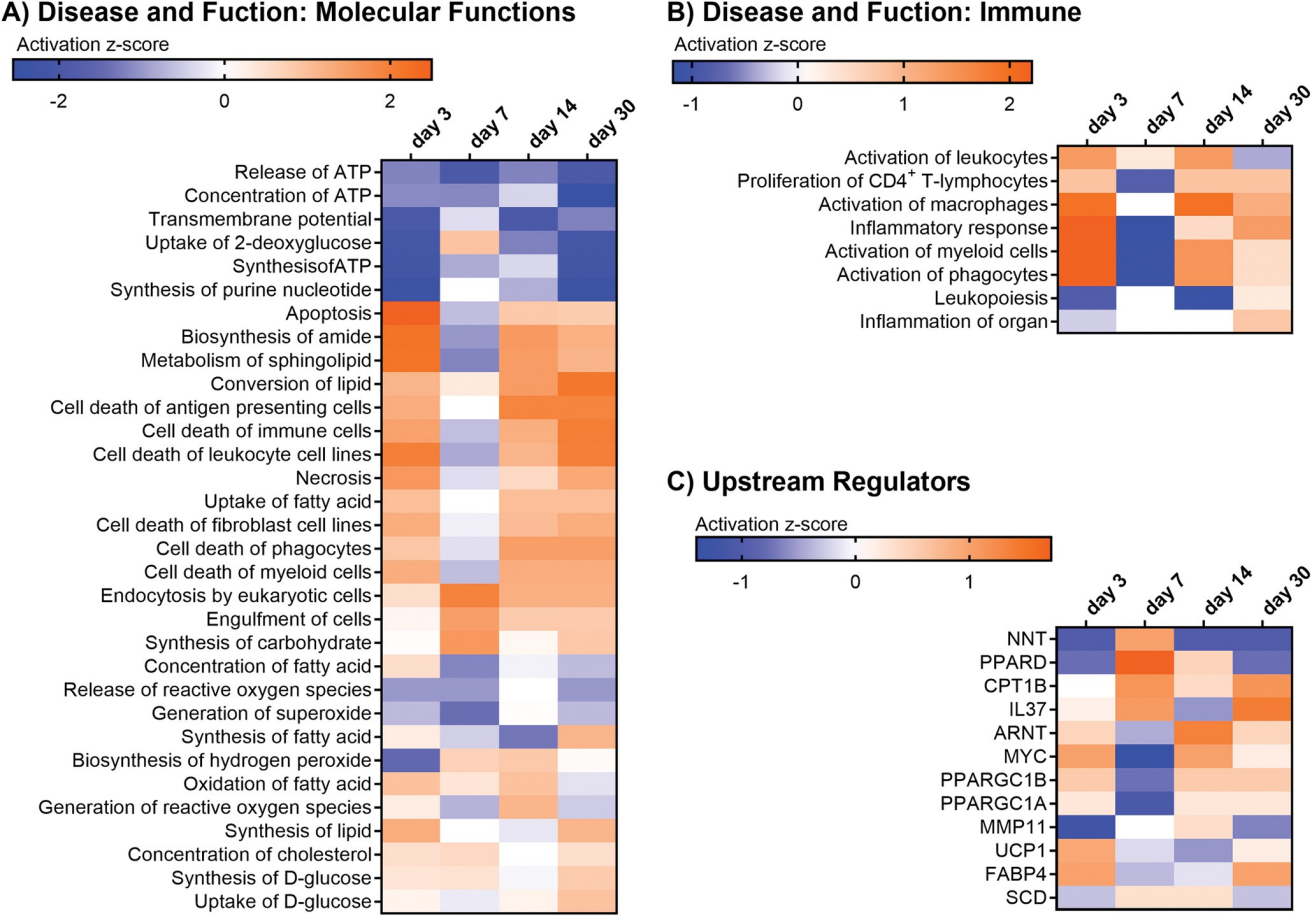
IPA pathway analysis of fecal metabolites pre- and post-vaccination. Identified Disease and Functions pathways for Molecular Functions (A) and Immune (B) pathways are shown to be significantly changed on days 3, 7, 14, and 30 post-vaccines. A z-score above 1.3 indicates significant upregulation (orange) when compared to day 0 (pre-vaccine). Conversely, a z-score below -1.3 indicates significant down regulation (blue). Upstream Regulators (C) identifies predicted global regulators that have been activated (orange) or inhibited (blue) which may explain the observed changes in metabolites.

To understand the significance of the metabolomic changes, we utilized Ingenuity Pathway Analysis (IPA). Metabolite data (**Table 1)** were entered as fold change in relation to data collected from day 0 (pre-vaccine). The predicted disease and functions pathways identified within molecular functions showed an overall decrease in the ATP production, as seen in the pathways ‘Release of ATP’, ‘Concentration of ATP’, and ‘Synthesis of ATP’ (**Fig 7A**). Upregulated pathways involved cell death (Apoptosis, Necrosis) on days 3, 14, and 30 post-vaccines but these decreased on day 7 (**S6A Fig**). A potential activation of immune cell functions (Endocytosis by eukaryotic cells, Engulfment of cells) was noticed with an increased activity at all days post vaccination. Other pathways showed a trending decrease in ‘Release of reactive oxygen species’ and ‘Generation of superoxide’ or a trending increase in ‘Synthesis of carbohydrate’, ‘Concentration of cholesterol’, ‘Synthesis of D-glucose’, and ‘Biosynthesis of hydrogen peroxide’ (**S6A Fig**).

Further analysis of immune-focused diseases and functions was performed (**Fig 7B)**. The ‘Activation of leukocytes’ was increased on days 3, 7, and 14 post-vaccines but decreased on day 30. Other pathways, such as ‘Inflammatory response’, ‘Activation of phagocytes’, ‘Activation of macrophages’, ‘Activation of myeloid cells’ and ‘Proliferation of CD4+ T-lymphocytes were increased on all days except day 7. Finally, in the first 14 days, ‘Leukopoiesis’ (formation of white blood cells) and ‘Inflammation of organ’ were decreased. This suggests a vaccine-associated activation of the immune system (**S6B Fig**).

The metabolite data were processed to determine if the changes of global regulators could be predicted. Using IPA Upstream Regulator Analysis, we identified proteins that are predicted to be activated or inhibited (**Fig 7C**). Of note, the anti-inflammatory cytokine IL-37 [30] is predicted to be activated days 3, 7, and 30 post-vaccination. Also, different regulators of mitochondria and metabolism were predicted to be upregulated on day 7 including NAD(P) transhydrogenase (NNT: mitochondria redox) [31], lipid-sensing peroxisome proliferator-activated receptor delta (PPARD) [32], and long-chain fatty acid transport carnitine palmitoyltransferase I (CPT1B) [33]. While both NNT and PPARD were downregulated on days 3 and 30. Other transcriptomic co-factors and regulators, such as ARNT (aryl hydrocarbon receptor nuclear translocator) [34], MYC [35] and PPARGC1 (Peroxisome proliferator-activated receptor gamma coactivator 1) [36], were predicted to be upregulated on days 3, 14 and 30 post-vaccination. Uncoupling protein 1 (UCP1) [37] growth factor and fatty acid binding protein 4 (FABP4) [38] were upregulated on days 3 and 30 but downregulated on days 7 and 14 post-vaccination. Matrix metalloproteinase 11 (MMP11) [39], an inflammatory mediator, and stearoyl-CoA desaturase (SCD) [40], an enzyme that participates in the synthesis of UFA from SFA, were downregulated on days 3 and 30 post-vaccination (**S6C Fig**). Overall, this analysis indicated a significant change in metabolism, inflammation and proliferation following vaccine administration.

## Discussion

An oral antigen-specific vaccine using live attenuated *Salmonella* prevented and reversed T1D in NOD mice [5–7]. To better understand possible effects of the vaccine, changes in the composition of the gut microbiota were determined at different time points following vaccination. This is important because the microbiota can protect the host against microbial pathogens [41] while effects of *Salmonella* infection on the gut microbiota may lead to inflammation that favors the growth of pathogens [42, 43]. And while the *Salmonella* strain employed in the vaccine was attenuated, it still retained some infectious capacity.

Changes in the gut microbiota were implicated in altering diabetes susceptibility in NOD mice [44, 45]. Related to this, changes in the gut microbiome were seen between healthy and diabetic individuals mimicking to an extent data from NOD mice [46]. In this study, we compared the gut microbiome in vaccine- and vehicle-treated prediabetic mice. We found no changes in the composition of gut microbiota on the phylum level after vaccine administration except for a decrease in *Firmicutes*, and *Actinobacteria* on day 30 and increase in *Bacteroidetes* on day 30. Furthermore, no changes in the composition of bacteria genera between vaccine- and vehicle-treated mice were noted at any time points except an increase of *Anaeroplasma* and *Corprococcus* on days 7 and 14, respectively. Our vaccine combination caused an increase in the gut phylum *Proteobacteria*, the class *Gammaproteobacteria*, and the families *Pseudomonadaceae*, and *Enterobacteriaceae*. *Salmonella* genus belongs to *Enterobacteriaceae* (family), *Gammaproteobacteria* (class), and *Proteobacteria* (phylum) [12]. This could be important for the changes seen after vaccination. Overall, the data indicate that administration of the *Salmonella*-based vaccine altered the balance of the gut microbiota in prediabetic NOD mice. However, the modest changes noted before and after treatment within the same treatment-group (either vaccine group or vehicle group) were likely due to other factors such as type of food, handling, temperature, and age. The slight increase in family *Pseudomonadacea* may be attributed to an imbalanced gastrointestinal environment due to temporary changes in the composition of the gut microbiome [47].

Studying the changes in the composition of gut microbiota at short (7–14 days post vaccination) and longer times (30 days post-vaccination) reflects the expected lifecycle of the vaccine. Short term, there was an increase in only a few taxa by the vaccine, suggesting a successful infection and proliferation of the vaccine within the GALT. At day 30, the composition of microbiota mostly returned to its pre-vaccinated state except for an increase in the *Bacteroidetes* phylum and decrease in the *Firmicutes* and *Actinobacteria*. Narratively, this aligns with our data on the approximate 3-week clearance time of the vaccine. This suggests that the microbiota may have assumed a more tolerant state. When the vaccine successfully infiltrated the GALT, Treg abundance was increased throughout several lymphatic tissues especially on day 30 post-vaccination and this was associated with less beta cell injury [6, 7]. Of some possible relevance, gut *Bacteroidetes* were associated with increased Tregs [22]. Based on our data, we postulate that the microbiota responded to the vaccine and participated in immune regulation and induction of tolerance by increasing Tregs and regulatory cytokines and decreasing inflammatory cytokines [6, 7]. The intestinal epithelium plays an important role as a filter that translocates water, nutrients, and bio-reactive compounds from the intestine to the circulation [48]. Inflammation can increase the intestinal permeability to permit antigens to induce autoimmune response and promote the development of diabetes [49, 50].

In this study, the metabolic profiling of the vaccine-treated animals was determined and identified. Of the 60 identified metabolites, almost a third was associated with diabetes. The rest of the common metabolites were associated with digestion and were expected based on the diet of the mice. For example, amino acids (AA) including glycine and alanine are associated with glucose metabolism through promoting insulin secretion [51, 52]. Furthermore, elevated levels of valine, isoleucine, leucine, tyrosine, and phenylalanine are correlated with insulin intolerance and secretion suppression [53, 54]. Throughout, the levels of most of AAs showed high variance from day to day. However, by day 30 most of the AAs returned to pre-vaccination levels. Of note, multiple predicted Upstream Regulators were up or down regulated based on the combination of changes in amino acid concentrations (**Fig 7C**). For example, CPT1B was predicted to be upregulated because of the increase in leucine and valine, and the decrease in alanine, glycine, and uracil (**S6C Fig**). When MMP11 is active, it could up-regulate amino acids such as alanine, glycine, isoleucine, tyrosine, phenylalanine, and ethanolamine. Also, due to the combined effects of alanine and glycine, in association with butyric acid and succinic acid, the anti-inflammatory protein IL37 is predicted to be activated on all days observed except day 14 (**S6C Fig**).

Lactate is produced by gut bacteria under anaerobic conditions and is metabolized in the colon by lactate-utilizing bacteria to produce beneficial short-chain fatty acids (SCFAs) including butyrate and propionate. Lactate metabolites play an important role in the stability of the gut microbiome [55]. SCFA metabolites generated by gut microbiota exert systemic anti-inflammatory effects through involvement in the production of immunoglobulin A and immunosuppressive cytokines [56]. These SCFAs may have an effect on metabolic and immune response, and inflammatory disease [57, 58]. For example, changes in the ‘Biosynthesis of hydrogen peroxidase are due, in part, to the changes in butyric and succinic acids (**S6A Fig**). SCFAs such as acetate and propionate can influence insulin sensitivity and glucose tolerance via glycemic-mediated response. Propionate can decrease the fasting blood glucose, reduce gluconeogenesis, promote the utilization of glucose, and elevate glucagon-like peptide [59].

Butyrate maintains the integrity of the gut epithelium by inducing mucin synthesis [57]. NOD mice had lower abundance of SCFAs, especially butyrate, when compared to non-diabetic mice [60]. Significant elevations in 3-hydroxybutyric acid (β-hydroxybutyric acid, BHBA) is an indicator of diabetic ketoacidosis in T1D [61, 62]. In this study, our vaccine was associated with a decrease in BHBA (**Table 1**). However, in general, the *Salmonella*-based vaccine increased the levels of SCFAs in the feces of NOD mice.

Myristic, palmitic, and stearic acid are saturated fatty acids (SFAs) commonly found at high levels in individual with T1D [53]. SFAs stimulate the Toll-like receptor 4 (TLR-4) to increase pro-inflammatory cytokine expression [63]. Conversely, dicarboxylic acids with fatty acid substituents like succinic acid stimulated insulin biosynthesis [64], increased the secretion of pro-inflammatory cytokines such as TNFα and IL1β, and activated T cells [65, 66]. Herein, we found that vaccinated mice had decreased succinic acid (butanedioic acid). Medium and long chain unsaturated fatty acids such as azelaic, linoleic, oleic, octanoic, and pentadecanoic acid promote insulin secretion [67–70]. Also, these SFAs (succinic, palmitic, oleic, linoleic acids) were indirectly regulated by MYC (**S6C Fig**). Of note, inhibiting the medium and long chain fatty acid β-cell receptor (GPR40) impaired insulin secretion [71].

An increase in glutamate and a decrease in TCA metabolites were observed in diabetic NOD mice [72]. Our vaccinated mice showed a significant decrease in L-5-oxoproline (pyroglutamic acid) (**Table 1**). Vaccination was not associated with a change in the TCA metabolite, oxalic acid. Monoacylglycerol increased the glucagon-like peptide-1 (GLP-1) and gastric inhibitory polypeptide (GIP) levels following administration to the small intestine [73]. Our vaccine did not show effect on the abundance of monoacylglycerol (**Table 1**). Steroid metabolites in the gut of NOD mice were changed as a result of *Salmonella*-induced inflammation [74]. Steroids (cholesterol, coprostanol, stigmasterol, and campesterol) and eicosanoid can impact wound healing, sugar metabolism, and immune system regulation [74]. In fact, changes in cholesterol influenced multiple Disease and Functions pathways such as ‘Concentration of ATP’, ‘Biosynthesis of amide’, ‘Synthesis of lipid’ (**S6A Fig**), and ‘Activation of leukocytes’, ‘Activation of phagocytes’, and ‘Inflammation of organ’ (**S6B Fig**). Many of the metabolites assessed in the feces of vaccine-treated mice were altered over time. However, analysis was not obtained on samples from unvaccinated mice. Hence, it is unknown if the changes in the metabolome were secondary to the vaccine alone.

In summary, we demonstrated that an oral *Salmonella* vaccine did not adversely alter gut microbiota diversity and proportions. At 30 days post-vaccination, the results suggested a transition in microbiota towards a more tolerant composition as the Tregs increased in mucosal tissues. Metabolic analysis indicated that after vaccination changes in metabolism, inflammation, and proliferation occurred and this was associated with immune activation and increased regulatory and decreased inflammatory cytokines. These results support the oral administration of *Salmonella* vaccines as carriers for autoantigens and immunomodulators against T1D. Further studies to understand the mechanistic role of the microbiota in immune function and to relate the composition of the microbiota to oral vaccines are required.

## Supporting information

S1 Fig Variation in fecal bacteria between vaccinated and vehicle-treated NOD mice. Abundance percentage of the fecal bacteria at the phylum level (A), genus level (B), and species level (C) in vaccine- (n = 25) and vehicle-treated (n = 25) mice.
(TIF)

S2 Fig Variation in fecal mycoses between vaccinated and vehicle-treated NOD mice. Abundance percentage of the fecal mycoses at the phylum level (A), genus level (B), and species level (C) in vaccine- (n = 25) and vehicle-treated (n = 25) mice.
(TIF)

S3 Fig Variation in fecal bacteria between vaccinated and vehicle-treated NOD mice. Abundance in percentage of the fecal bacteria at (A) the phylum level, and (B) genus level in the feces of NOD mice at time points pre-, 3-, 7-, 14-, and 30-days post-vaccination with oral *Salmonella*-based vaccine.
(TIF)

S4 Fig Variation in fecal mycoses between vaccinated and vehicle-treated NOD mice. Abundance percentage of the fecal mycoses at (A) the phylum level, and (B) the genus level in NOD mice at different time points pre and 3-, 7-, 14-, and 30-days post-vaccination with oral *Salmonella*-based vaccine.
(TIF)

S5 Fig Boxplots of changes in abundance percentage of the fecal mycoses at the phylum and genus levels. (A) The 4 most abundant phyla including *Ascomycota*, *Basidiomycota*, *Zygomycota*, *and Glomeromycota*. (B) The 4 most abundant genera included *Galactomyce*s, *Eurotium*, *Candida*, and *Geotrichum*. Each time point represents the mean ± SD of 4–6 samples. Group of vaccinated mice compared with vehicle treated at different time points. Mann-Whitney with Holm-Sidak correction for multiple comparisons test was used to report significance between groups at each time point using asterisks, *p < 0.05.
(TIF)

S6 Fig The predicted pathways after vaccine administration. For each analysis, relevant metabolite measurements are located on the outside of the circle. The spokes leading to the center are the predicted relationship to the pathway which is in the center. Arrows on the spokes indicate that metabolite activates the pathway whereas bars indicate inhibition. **A) Disease and Functions: Molecular Functions**. Relative changes on days 3, 7, 14, and 30 in pathways Concentration of ATP, Biosynthesis of amide, Biosynthesis of hydrogen peroxide, and Synthesis of lipid. Legend of predicted measurements. **B) Disease and Functions: Immune**. Relative changes on days 3, 7, 14, and 30 are indicated in pathways Activation of leukocytes, Activation of phagocytes, and Inflammation of organ. Legend of predicted measurements. **C**) **Upstream Regulators**. Relative changes in CPT1B, IL37, MYC, and MMP11 on days 3, 7, 14, and 30 are indicated. Legend of predicted measurements.
(ZIP)
