# Supplementary figures and images for: Changes in the gut microbiota of NOD mice in response to an oral Salmonella-based vaccine against type 1 diabetes
Source: PLoS One. 2023 May 24;18(5):e0285905. doi: 10.1371/journal.pone.0285905 (PMC10208495; doi:10.1371/journal.pone.0285905)

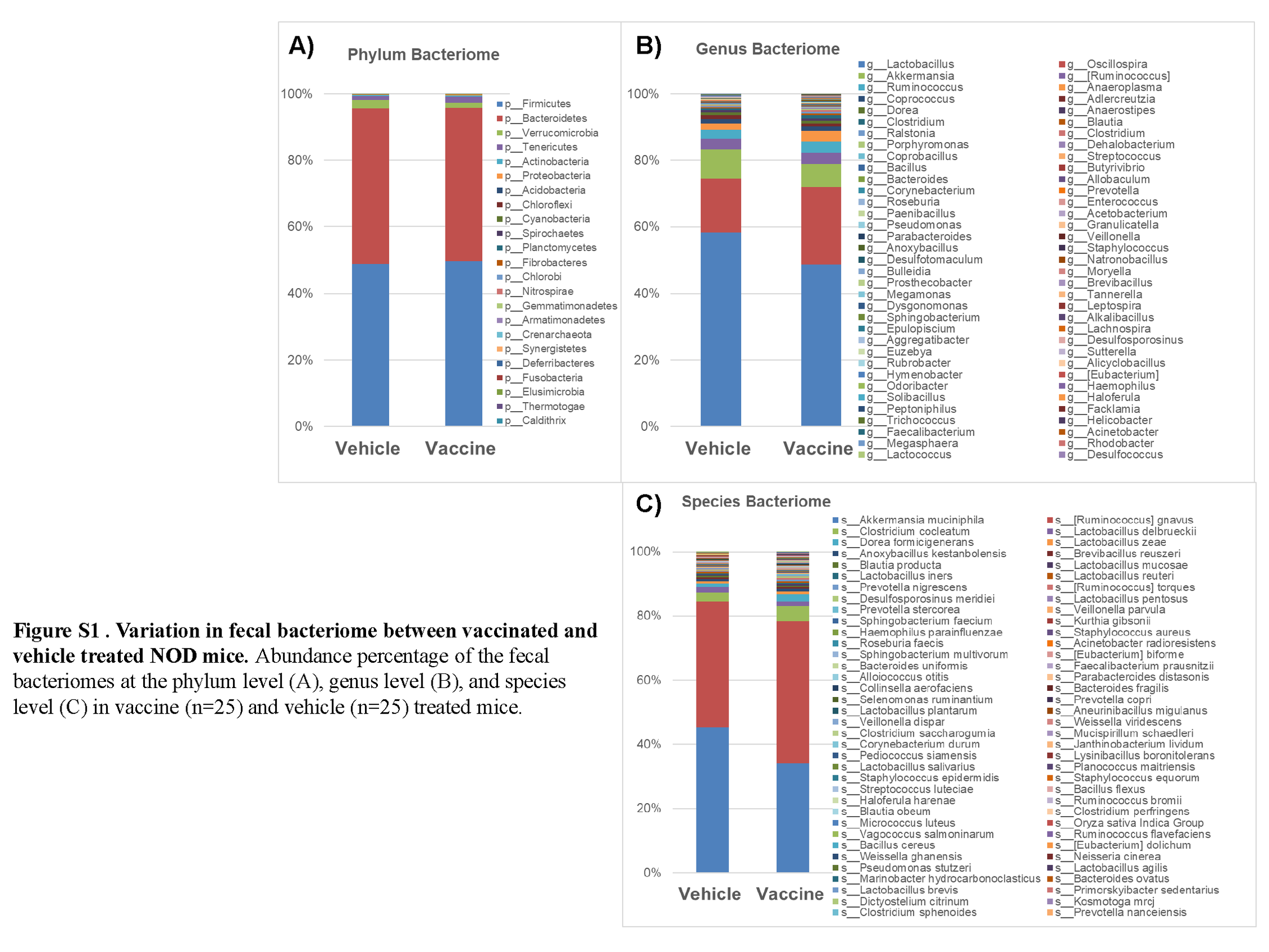

Supplement: S1 Fig — Abundance percentage of the fecal bacteria at the phylum level (A), genus level (B), and species level (C) in vaccine- (n = 25) and vehicle-treated (n = 25) mice. (TIF) [file pone.0285905.s001.tif]

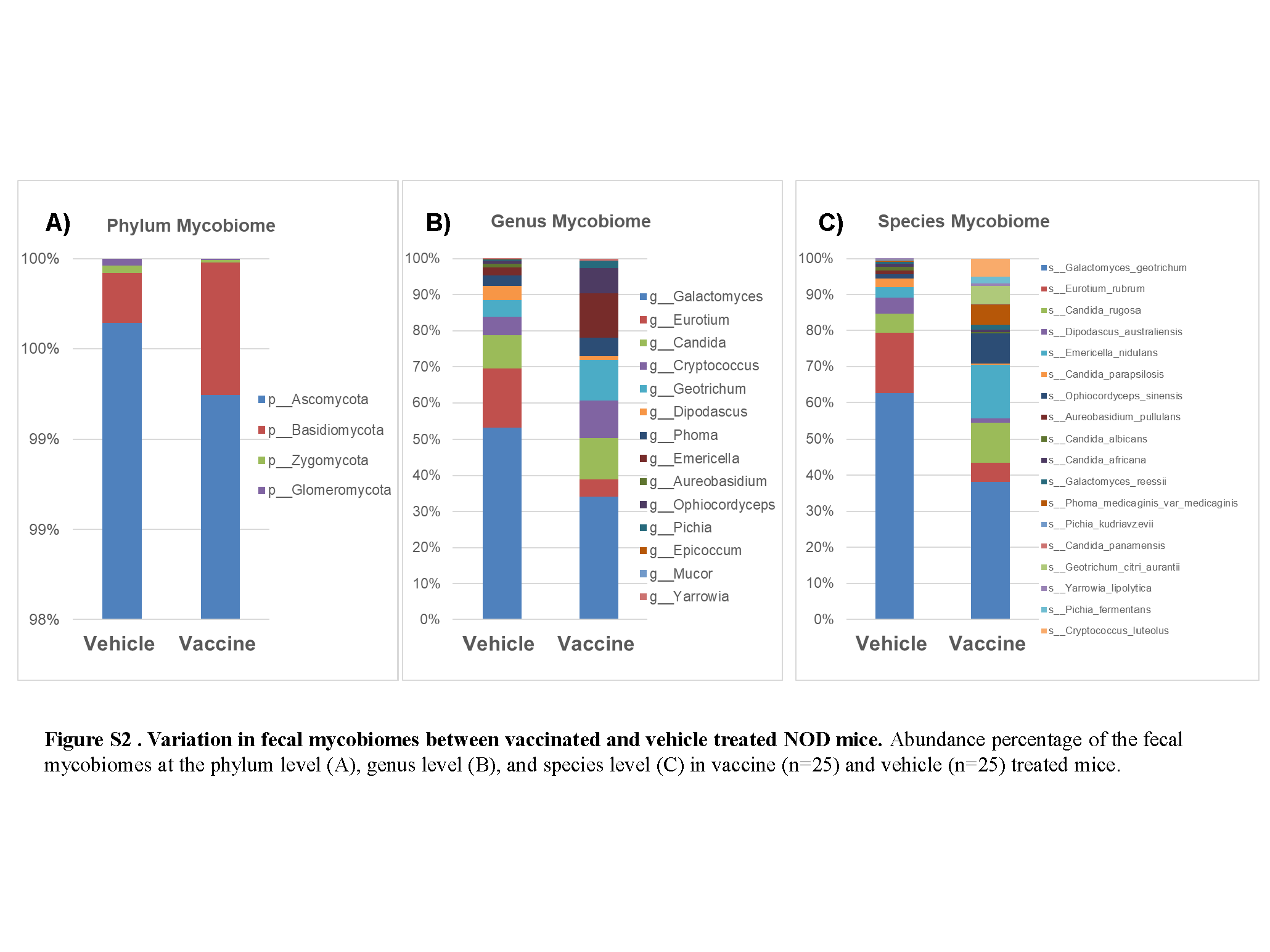

Supplement: S2 Fig — Abundance percentage of the fecal mycoses at the phylum level (A), genus level (B), and species level (C) in vaccine- (n = 25) and vehicle-treated (n = 25) mice. (TIF) [file pone.0285905.s002.tif]

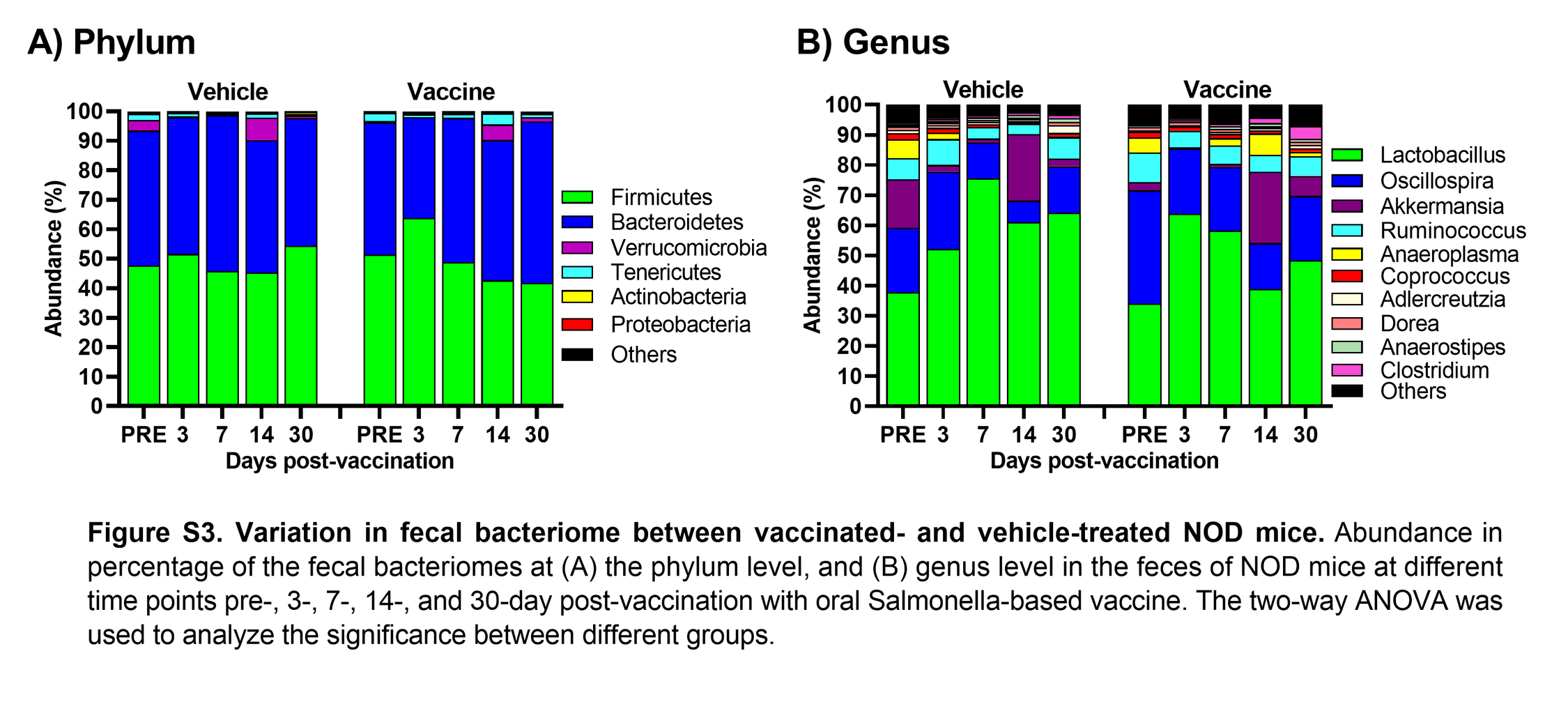

Supplement: S3 Fig — Abundance in percentage of the fecal bacteria at (A) the phylum level, and (B) genus level in the feces of NOD mice at time points pre-, 3-, 7-, 14-, and 30-days post-vaccination with oral Salmonella-based vaccine. (TIF) [file pone.0285905.s003.tif]

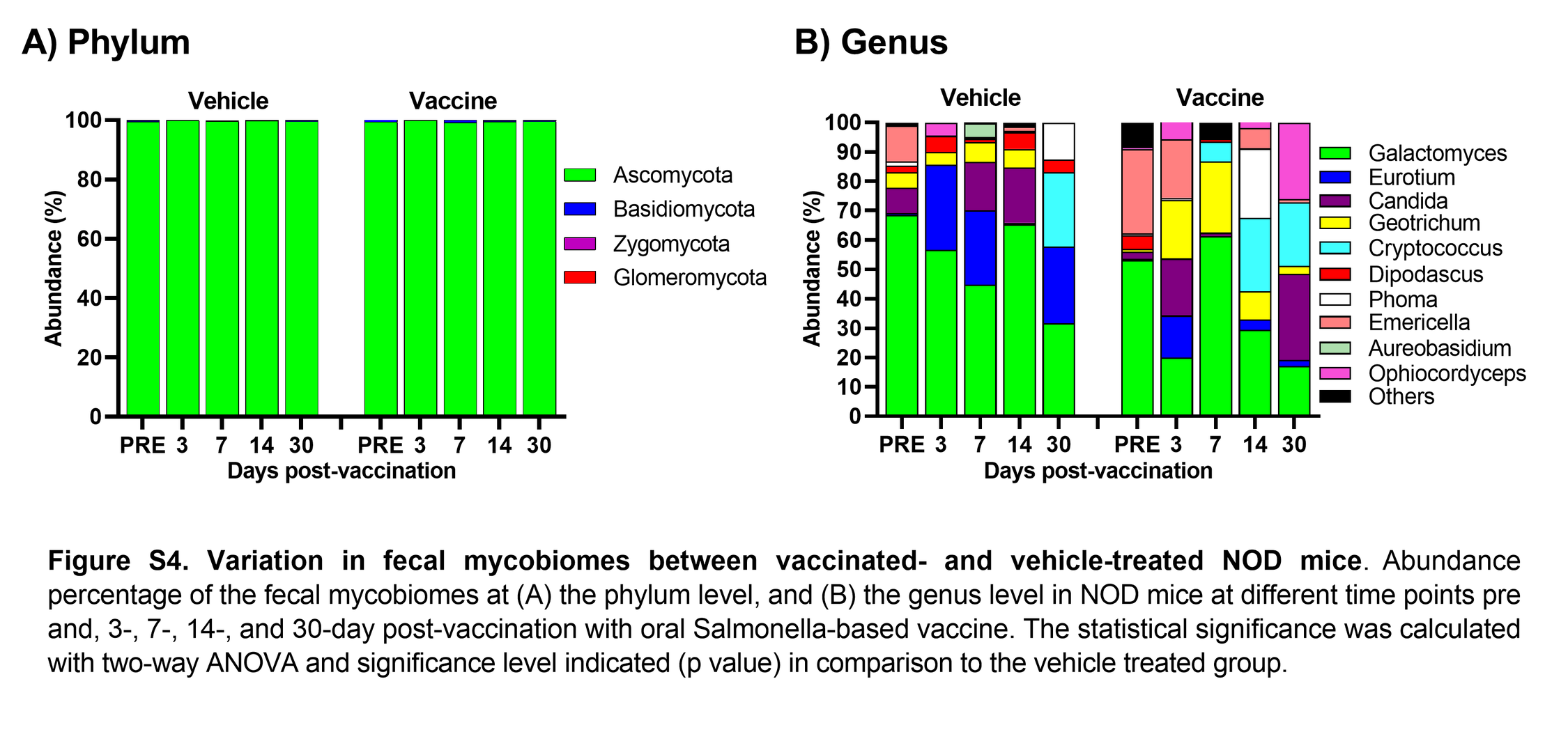

Supplement: S4 Fig — Abundance percentage of the fecal mycoses at (A) the phylum level, and (B) the genus level in NOD mice at different time points pre and 3-, 7-, 14-, and 30-days post-vaccination with oral Salmonella-based vaccine. (TIF) [file pone.0285905.s004.tif]

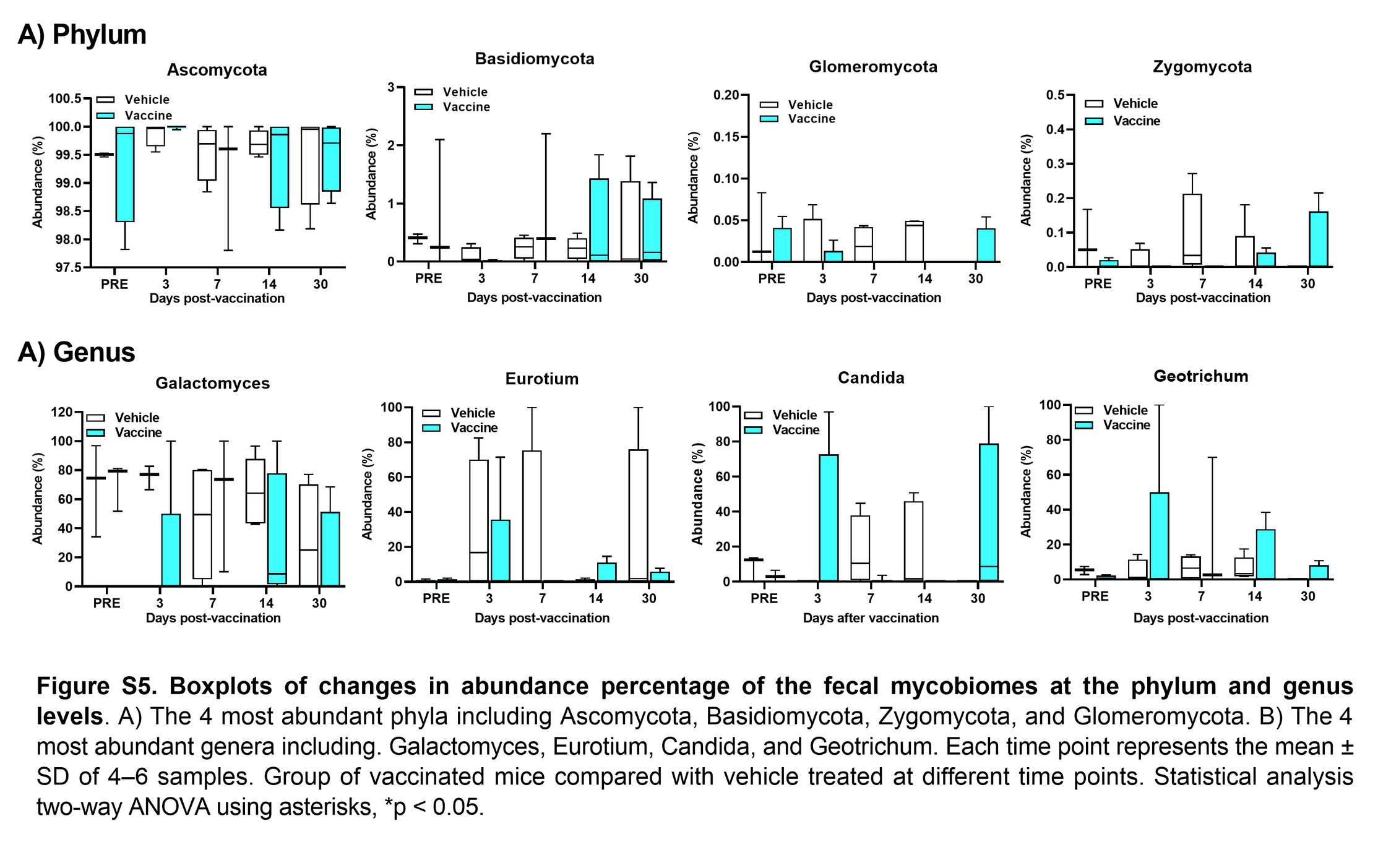

Supplement: S5 Fig — (A) The 4 most abundant phyla including Ascomycota, Basidiomycota, Zygomycota, and Glomeromycota. (B) The 4 most abundant genera included Galactomyces, Eurotium, Candida, and Geotrichum. Each time point represents the mean ± SD of 4–6 samples. Group of vaccinated mice compared with vehicle treated at different time points. Mann-Whitney with Holm-Sidak correction for multiple comparisons test was used to report significance between groups at each time point using asterisks, *p < 0.05. (TIF) [file pone.0285905.s005.tif]

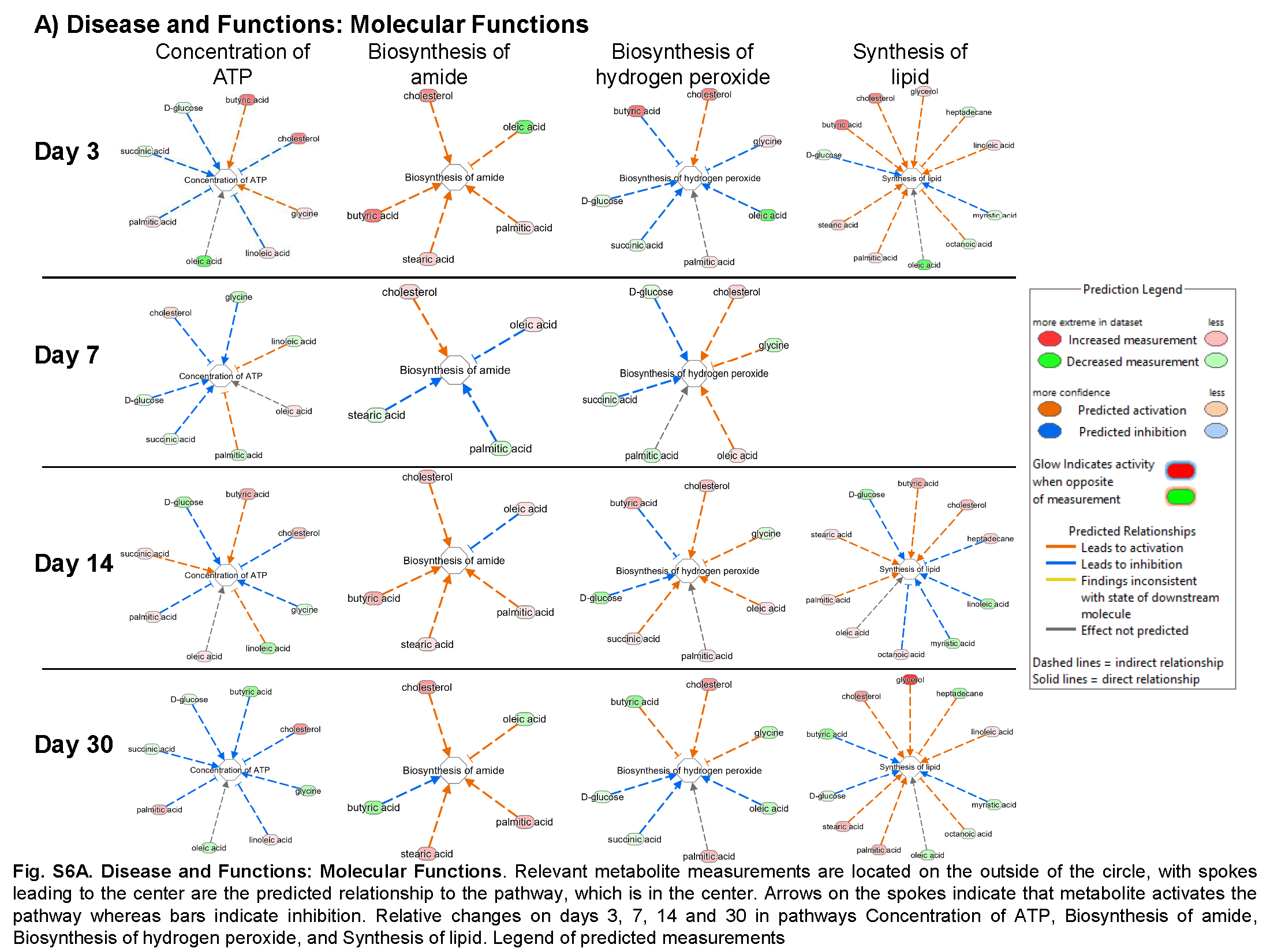

Supplement: S6 Fig — For each analysis, relevant metabolite measurements are located on the outside of the circle. The spokes leading to the center are the predicted relationship to the pathway which is in the center. Arrows on the spokes indicate that metabolite activates the pathway whereas bars indicate inhibition. A) Disease and Functions: Molecular Functions. Relative changes on days 3, 7, 14, and 30 in pathways Concentration of ATP, Biosynthesis of amide, Biosynthesis of hydrogen peroxide, and Synthesis of lipid. Legend of predicted measurements. B) Disease and Functions: Immune. Relative changes on days 3, 7, 14, and 30 are indicated in pathways Activation of leukocytes, Activation of phagocytes, and Inflammation of organ. Legend of predicted measurements. C) Upstream Regulators. Relative changes in CPT1B, IL37, MYC, and MMP11 on days 3, 7, 14, and 30 are indicated. Legend of predicted measurements. (ZIP) [file pone.0285905.s006.zip › S6A_Fig.tif]

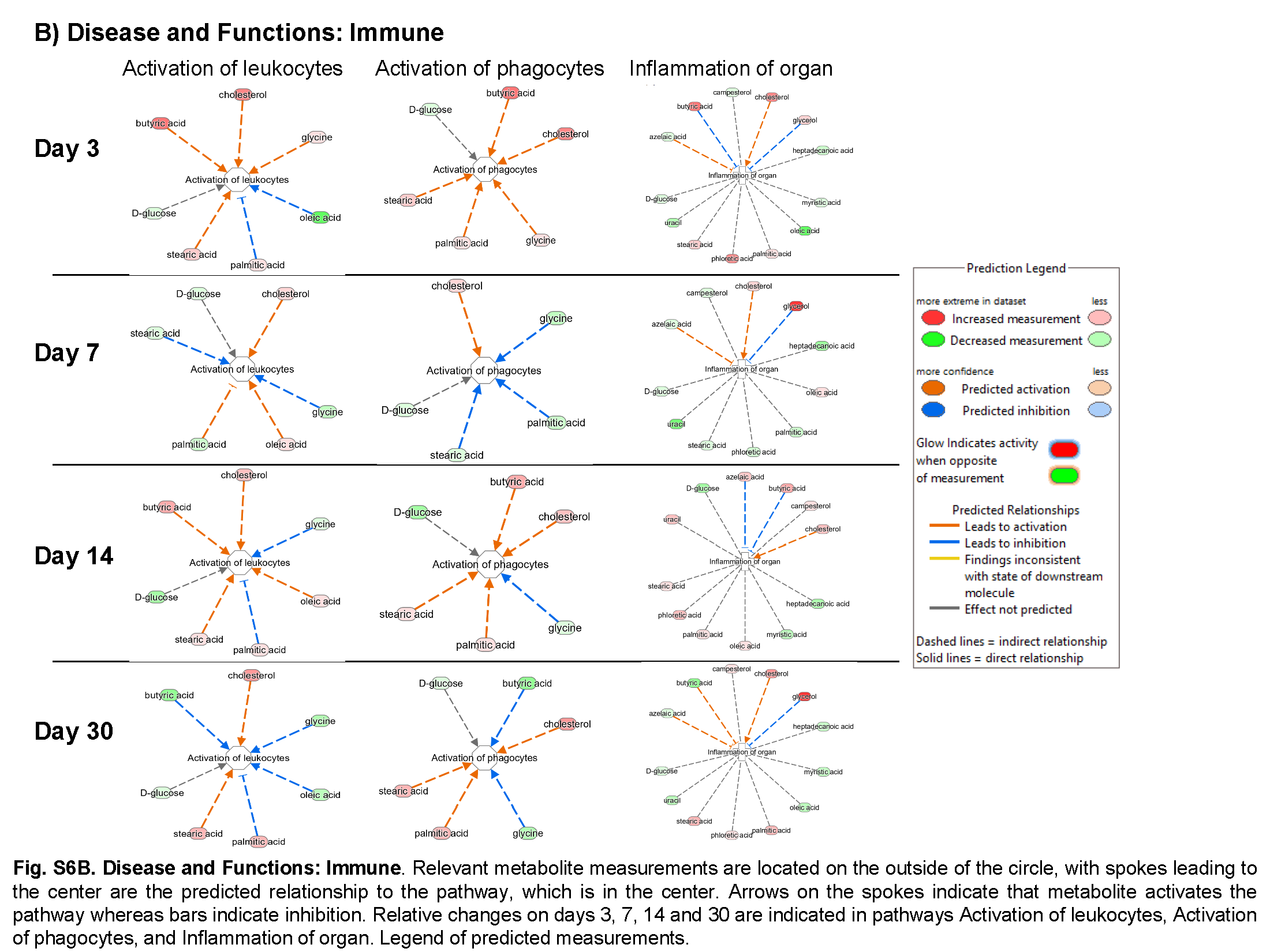

Supplement: S6 Fig — For each analysis, relevant metabolite measurements are located on the outside of the circle. The spokes leading to the center are the predicted relationship to the pathway which is in the center. Arrows on the spokes indicate that metabolite activates the pathway whereas bars indicate inhibition. A) Disease and Functions: Molecular Functions. Relative changes on days 3, 7, 14, and 30 in pathways Concentration of ATP, Biosynthesis of amide, Biosynthesis of hydrogen peroxide, and Synthesis of lipid. Legend of predicted measurements. B) Disease and Functions: Immune. Relative changes on days 3, 7, 14, and 30 are indicated in pathways Activation of leukocytes, Activation of phagocytes, and Inflammation of organ. Legend of predicted measurements. C) Upstream Regulators. Relative changes in CPT1B, IL37, MYC, and MMP11 on days 3, 7, 14, and 30 are indicated. Legend of predicted measurements. (ZIP) [file pone.0285905.s006.zip › S6B_Fig.tif]

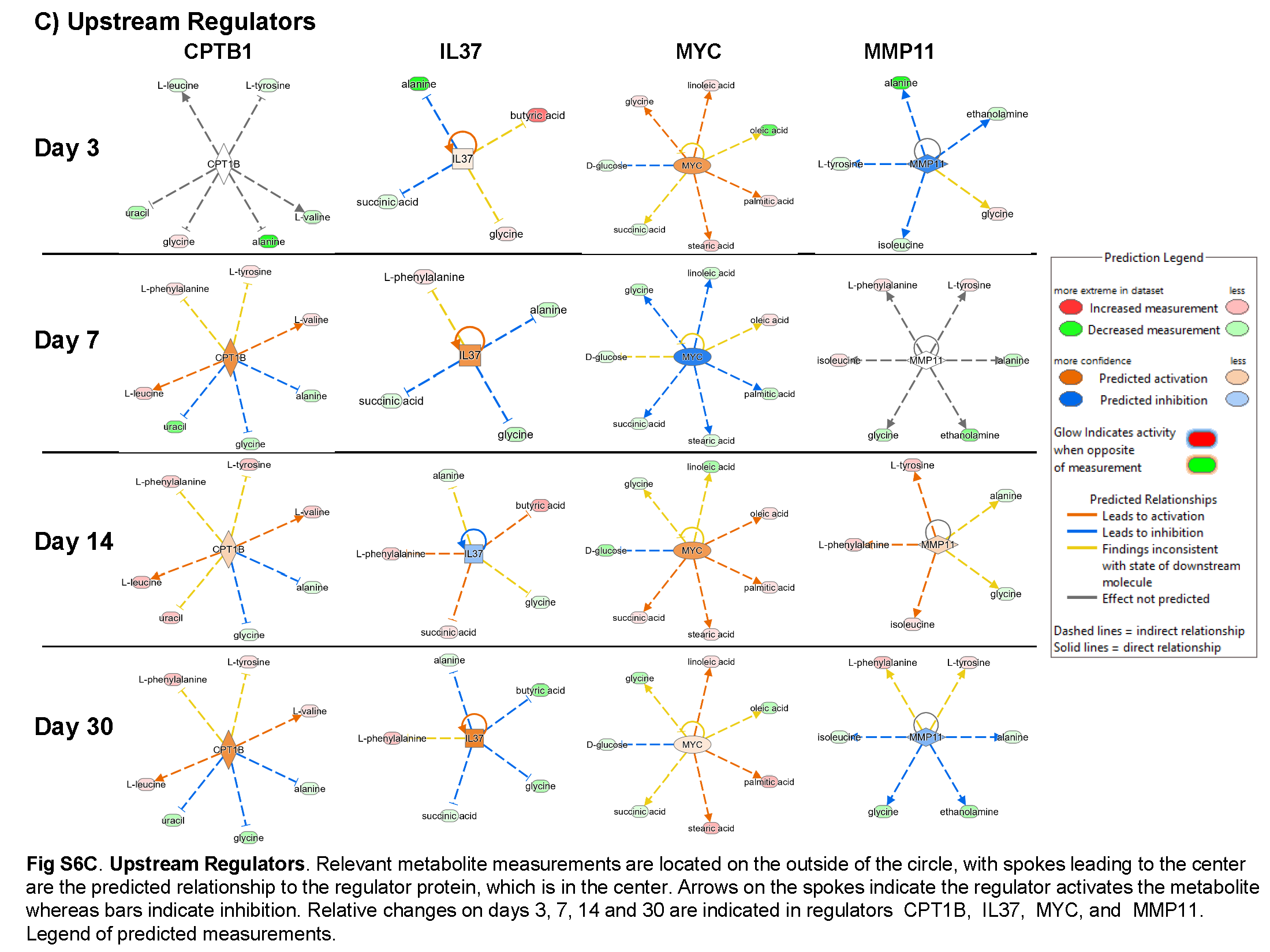

Supplement: S6 Fig — For each analysis, relevant metabolite measurements are located on the outside of the circle. The spokes leading to the center are the predicted relationship to the pathway which is in the center. Arrows on the spokes indicate that metabolite activates the pathway whereas bars indicate inhibition. A) Disease and Functions: Molecular Functions. Relative changes on days 3, 7, 14, and 30 in pathways Concentration of ATP, Biosynthesis of amide, Biosynthesis of hydrogen peroxide, and Synthesis of lipid. Legend of predicted measurements. B) Disease and Functions: Immune. Relative changes on days 3, 7, 14, and 30 are indicated in pathways Activation of leukocytes, Activation of phagocytes, and Inflammation of organ. Legend of predicted measurements. C) Upstream Regulators. Relative changes in CPT1B, IL37, MYC, and MMP11 on days 3, 7, 14, and 30 are indicated. Legend of predicted measurements. (ZIP) [file pone.0285905.s006.zip › S6C_Fig.tif]
